# Causal relationship of cereal intake and type with cardiovascular disease: a Mendelian randomization study

**DOI:** 10.3389/fnut.2023.1320120

**Published:** 2024-01-23

**Authors:** Jianhui Liu, Dihui Cai

**Affiliations:** Department of Cardiology, Ningbo Medical Center of Lihuili Hospital, Ningbo, Zhejiang, China

**Keywords:** Mendelian randomization, cardiovascular disease, cereals, muesli, body mass index

## Abstract

**Background:**

Observational studies have suggested that cereal consumption is associated with a reduced risk of cardiovascular disease (CVD). However, the potential causal relationship is not clear. We aimed to investigate the association of cereal intake and cereal type with CVD risk.

**Methods:**

Two-step Mendelian randomization (MR) analysis was performed to confirm the causal association of cereal intake and cereal type with the risk of several common CVDs. Furthermore, two-step MR analysis was used to explore the mediating effect of cardiovascular metabolic factors, and multivariable MR analysis was used to assess the impact of socioeconomic status, such as education and income, on the causal association.

**Results:**

The MR analysis indicated that genetically predicted cereal intake is associated with reduced risk of large artery stroke (LAS) (odd ratio (OR): 0.421; 95% confidence interval (CI) [0.193, 0.918]; *p* = 0.030), and muesli as the primary cereal intake is associated with reduced risk of coronary heart disease (CHD) (OR: 0.100; 95% CI [0.023, 0.437]; *p* = 0.002), myocardial infarction (MI) (OR: 0.101; 95% CI [0.020, 0.509]; *p* = 0.005), heart failure (OR: 0.210; 95% CI [0.064, 0.684]; *p* = 0.010), ischemic stroke (IS) (OR: 0.130; 95% CI [0.029, 0.591]; *p* = 0.008), LAS (OR: 0.017; 95% CI [0.0004, 0.737]; *p* = 0.034), and small-vessel stroke (OR: 0.021; 95% CI [0.001, 0.708]; *p* = 0.005). In contrast, genetically predicted biscuits as the primary cereal intake increased the risk of CHD (OR: 6.557; 95% CI [1.197, 36.031]; *p* = 0.031), and other cereals, such as cornflakes, as the primary cereal intake increased the risk of CHD (OR: 3.803; 95% CI [1.194, 12.111]; *p* = 0.024), MI (OR: 4.240; 95% CI [1.185, 15.174]; *p* = 0.026), stroke (OR: 3.154; 95% CI [1.070, 9.298]; *p* = 0.037), and IS (OR: 3.736; 95% CI [1.185, 11.782]; *p* = 0.024). Multivariable MR analysis underscored the significant role of education and income in the causal association, and two-step MR analysis indicated that body mass index, lipids, and blood glucose exerted important mediating effects in the causal association.

**Conclusion:**

The findings of our study underscore the causal beneficial influence of muesli as the primary cereal intake on CVDs. A reasonable consumption of muesli may provide primary prevention of CVDs.

## 1 Introduction

Cardiovascular diseases (CVDs), defined as a spectrum of disorders affecting the heart and blood vessels (1, 2), remain the leading cause of chronic death worldwide and represent a significant and enduring challenge to economies and healthcare systems (3). In 2020 alone, CVDs were responsible for approximately 19 million deaths worldwide, marking an 18.7% increase in a decade (4). Early prevention and intervention through the identification of risk factors can help reduce the incidence and progression of CVDs (5).

Implementing population-based health strategies that target modifiable risk factors, including adopting a healthy diet, can effectively prevent CVDs (6). Breakfast cereals, made from grains such as oats, corn, wheat, or rice, are a commonly consumed food at breakfast. The production of breakfast cereals typically involves minimal processing (e.g., rolling and drying) or more substantial processing (e.g., cooking, flaking, or puffing), with the possibility of mixing multiple grains and adding nuts and fruits. Breakfast cereals are usually consumed with milk or yogurt, but they can also be eaten dry (7). Observational studies have shown that cereal consumption has a protective effect against the development of CVD (8) and its risk factors, including high cholesterol levels (9), obesity (7), and type 2 diabetes (10).

While there is mounting evidence to suggest that cereal consumption can provide protection against CVDs, it remains unclear whether the protective effect is solely due to cereal intake. As most of the existing findings come from observational studies, the impact of confounding factors and reverse causality cannot be overlooked (11). Therefore, it is crucial to investigate the causality between cereal intake and CVD to better prevent potential CVD risks.

Causality between a trait and an outcome can be validated by Mendelian randomization (MR) analysis, which employs genetic variation as instrumental variables (IVs) (12). Due to the random distribution of genetic variation at birth, MR analysis effectively mitigates potential confounders and reverses the causation that often plagues observational studies, thereby strengthening causal inference (13).

To date, there have been no MR studies that have specifically investigated the causality of cereal intake and type on the incidence of CVD. This study aims to evaluate the potential causality of cereal intake and cereal type on various CVDs using a two-sample MR analysis.

## 2 Methods

### 2.1 Study design and data sources

**Figure 1:**
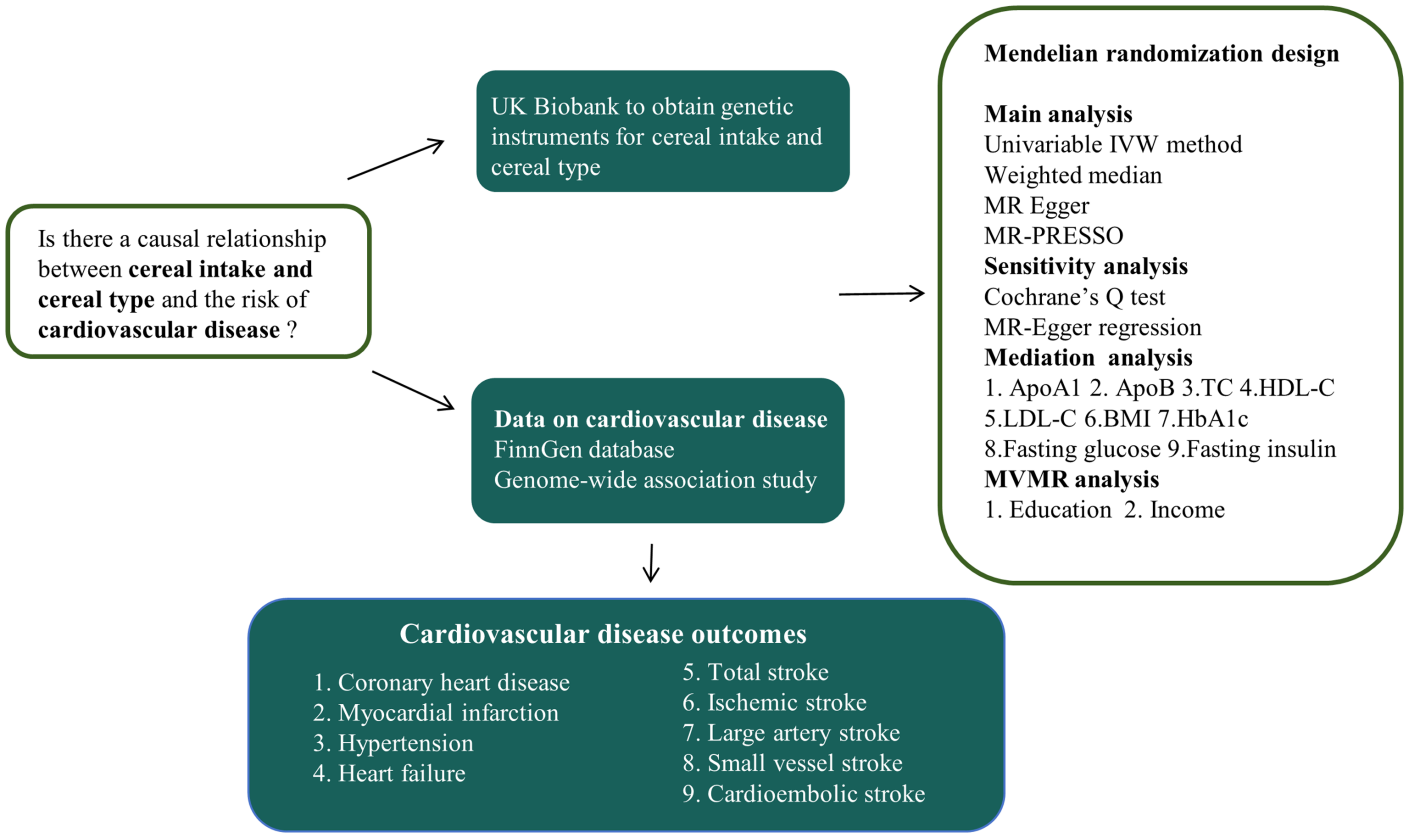
Overview of the study design. IVW, inverse-variance weighting; MR-PRESSO, MR pleiotropy residual sum and outlier test; ApoA1, apolipoprotein A-I; ApoB, apolipoprotein B; LDL-C, low-density lipoprotein total cholesterol; HDL-C, high-density lipoprotein total cholesterol; BMI, body mass index; TC, total cholesterol; HbA1c, glycosylated hemoglobin; MVMR, multivariable Mendelian randomization.

**Figure 2:**
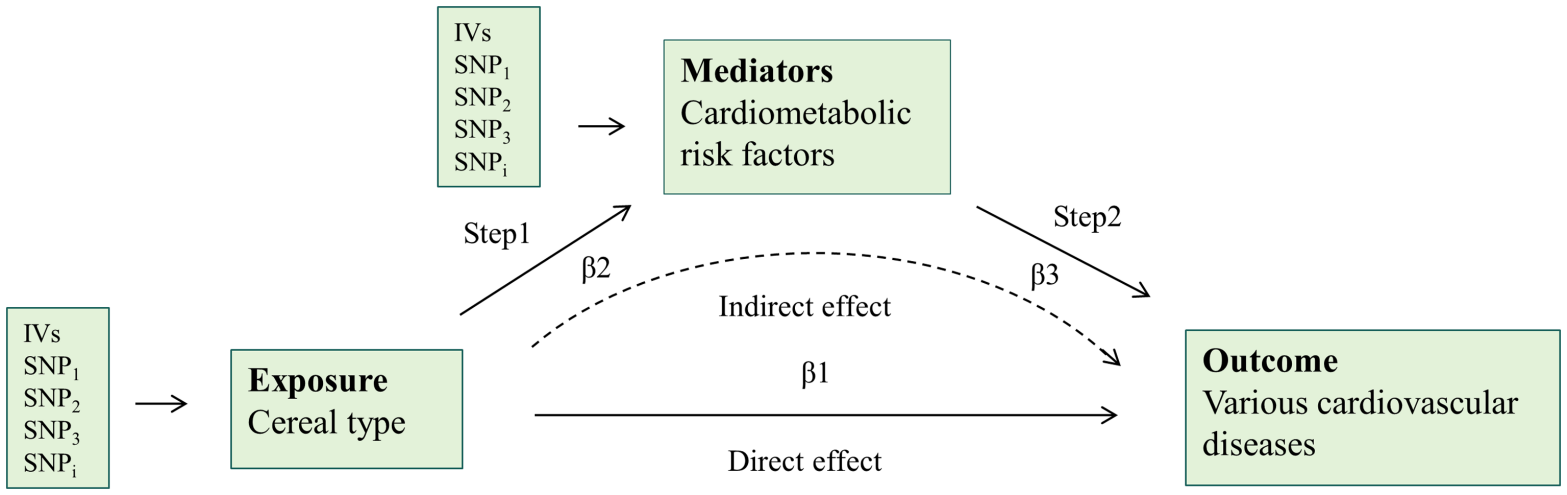
Overview of the two-step mediation analysis of cereal type on cardiovascular disease via potential mediators. Direct effect = β1–β2*β3; Indirect effect = β2*β3. IVs, instrumental variables.

An overview of the study design is shown in Figure 1. First, two-sample MR analyses were conducted to examine the causal association between cereal intake and cereal type and various CVD risks. Subsequently, we conducted a mediation analysis to identify potential cardiovascular metabolic mediators between cereal type and CVD (Figure 2). Finally, considering the impact of socioeconomic status (SES) on dietary habits, multivariable MR (MVMR) was used to examine the causal association after adjusting for education and income.

In this research, the causal relationship was evaluated using single-nucleotide polymorphisms (SNPs) as instrumental variables (IVs). The MR analysis was conducted under the following three key assumptions: (a) the IVs are strongly associated with the exposure variables; (b) the IVs are not related to confounding factors; (c) the IVs affect the risk of the outcomes only through the exposure (14).

Summary-level data on cereal intake and type were obtained from the UK Biobank. The cereal intake of participants was collected by a questionnaire asking about the frequency of cereal intake: “How many bowls of cereal do you eat per week?.” Cereal-type data was collected from all participants except those who reported no cereal or less than one bowl of cereal per week. Then, these participants were asked, “What type of cereal do you mainly eat?”

Additionally, data on various CVDs and their risk factors, such as coronary heart disease (CHD), myocardial infarction (MI), hypertension (HTN), heart failure (HF), stroke, ischemic stroke (IS), large artery stroke (LAS), small vessel stroke (SVS), and cardioembolic stroke (CES), as well as body mass index (BMI), low-density lipoprotein cholesterol (LDL-C), high-density lipoprotein cholesterol (HDL-C), apolipoprotein A-I, apolipoprotein B, total cholesterol (TC), fasting glucose (FG), fasting insulin (FI), and glycosylated hemoglobin (HbA1c), were obtained from the FinnGen database and other publicly available genome-wide association studies (GWAS). Table 1 provides a comprehensive list of GWAS for all exposures, outcomes, and mediators.

**Table 1:** Data sources.

Phenotypes	Data source	Phenotypic code	Cases/controls	Ancestry
Exposures				
Cereal intake	UK Biobank	ukb-b-15926	441,640	European
Bran cereal (e.g., All Bran, Branflakes)	UK Biobank	ukb-d-1468_1	50,609/249,289	European
Biscuit cereal (e.g., Weetabix)	UK Biobank	ukb-d-1468_2	53,310/246,588	European
Oat cereal (e.g., Ready Brek, porridge)	UK Biobank	ukb-d-1468_3	76,351/223,547	European
Muesli	UK Biobank	ukb-d-1468_4	61,523/238,375	European
Other (e.g., Cornflakes, Frosties)	UK Biobank	ukb-d-1468_5	58,105/241,793	European
Education	SSGAC	ieu-a-1239	766,345	European
Household income	UK Biobank	ukb-b-7408	397,751	European
Outcome				
Coronary artery disease	CARDIoGRAMplusC4D	ieu-a-7	60,801/123,504	77% European
Myocardial infarction	CARDIoGRAMplusC4D	ieu-a-798	43,676/128,199	77% European
Heart failure	HERMES	ebi-a-GCST009541	47,309/930,014	European
Hypertension	FinnGen	finn-b-I9_HYPTENS	55,917/162,837	European
Total Stroke	MEGASTROKE	ebi-a-GCST006906	40,585/406,111	European
Ischemic stroke	MEGASTROKE	ebi-a-GCST006908	34,217/406,111	European
Large artery stroke	MEGASTROKE	ebi-a-GCST006907	4,373/406,111	European
Small vessel stroke	MEGASTROKE	ebi-a-GCST006909	5,386/406,111	European
Cardioembolic stroke	MEGASTROKE	ebi-a-GCST006910	7,193/406,111	European
Mediator				
BMI	Hoffmann et al.	ebi-a-GCST006368	315,347	European
LDL-C	Kettunen et al.	met-c-895	21,559	European
HDL-C	Kettunen et al.	met-c-864	21,555	European
Serum total cholesterol	Kettunen et al.	met-c-933	21,491	European
Apolipoprotein A-I	Kettunen et al.	met-c-842	20,687	European
Apolipoprotein B	Kettunen et al.	met-c-843	20,690	European
HbA1c	Within family GWAS consortium	ieu-b-4842	45,734	European
Fasting glucose	Chen J et al.	ebi-a-GCST90002232	200,622	European
Fasting insulin	Chen J et al.	ebi-a-GCST90002238	151,013	European

BMI, body mass index; LDL-C, low-density lipoprotein total cholesterol; HDL-C, high-density lipoprotein total cholesterol; HbA1c, glycosylated hemoglobin.

### 2.2 Selection of instrumental variables

Genetic variations with a threshold below *p* < 5 × 10^−8^ were deemed to have statistical significance. Linkage disequilibrium analyses were conducted to assess the existence of linkage disequilibrium (*r*^2^ < 0.001; distance threshold, 10,000 kb). PhenoScanner (15) was utilized to search for the used SNPs and to assess their association with potential confounders, such as hypertension, BMI, diabetes, smoking, cholesterol, and insomnia, and SNPs that exhibited a strong association with the confounding factors (*p* < 5 × 10^−8^) were excluded. Supplementary Tables S1–S6 displays the information regarding the utilized SNPs for all exposures. F-statistics were employed to account for the issue of weak instrument bias in the selected SNPs. An F-statistic >10 was considered sufficient to establish a strong correlation between the IVs and the exposure, thereby avoiding the problem of weak instrument bias (16).

### 2.3 Statistical analyses

R software (version 4.2.0) and R packages like TwoSampleMR and MR-PRESSO were utilized to conduct statistical analyses. In this study, we performed various MR analysis methods to estimate the causality of cereal intake and cereal type on different CVDs. The inverse-variance weighting (IVW) method was used as the main method in MR analyses because the results of this method were generally the most reliable when the total IVs were valid (13). We also used MR-pleiotropy residual sum and outlier (MR-PRESSO) to detect and correct for outliers in the IVW linear regression. MR-Egger (17) and weighted median (18) analyses were additionally conducted as secondary supplements. Sensitivity analyses were performed to further evaluate the validity of the causal effect. Cochrane’s *Q* test was used to evaluate heterogeneity of SNPs, and when significant heterogeneity was present (*p* < 0.05), the multiplicative random effects IVW method was used as the primary approach to achieve a conservative and robust estimate. The Bonferroni correction method was used to identify false-positive results caused by multiple tests. *p* < 0.0056 (0.05/9) were considered statistically significant, while associations with *p* > 0.005 and *p* < 0.05 were defined as suggestive associations. Additionally, MR-Egger regression and the MR-PRESSO global test were employed to assess the presence of directional pleiotropy among the IVs.

### 2.4 Mediation MR analysis

Figure 2 illustrates the design of the mediation analysis. Obesity, glycometabolism, and lipid metabolism are associated with cereal consumption and CVD. We utilized a two-step methodology to evaluate the extent to which CVD risk factors mediate the causal effect. First, after excluding SNPs strongly related to CVD risk factors mentioned in the Selection of Instrumental Variables, we assessed the causality between cereal type and these potential mediators using IVs strongly related to cereal type. Second, the IVs strongly linked to the mediators were utilized to estimate the impact of the mediators on various CVD risks adjusted for the specific cereal type. Direct effect = β1–β2*β3 and indirect effect = β2*β3.

## 3 Results

### 3.1 Selection of instrumental variables

Detailed information on the SNPs used to predict cereal intake and type is displayed in Supplementary Tables S1–S6. All SNPs satisfied the screening criteria in the Methods section, and the F-statistic of SNP > 10 indicates no weak instrument bias.

### 3.2 Causal estimates of genetic susceptibility to cereal intake and type of CVD risk

**Figure 3:**
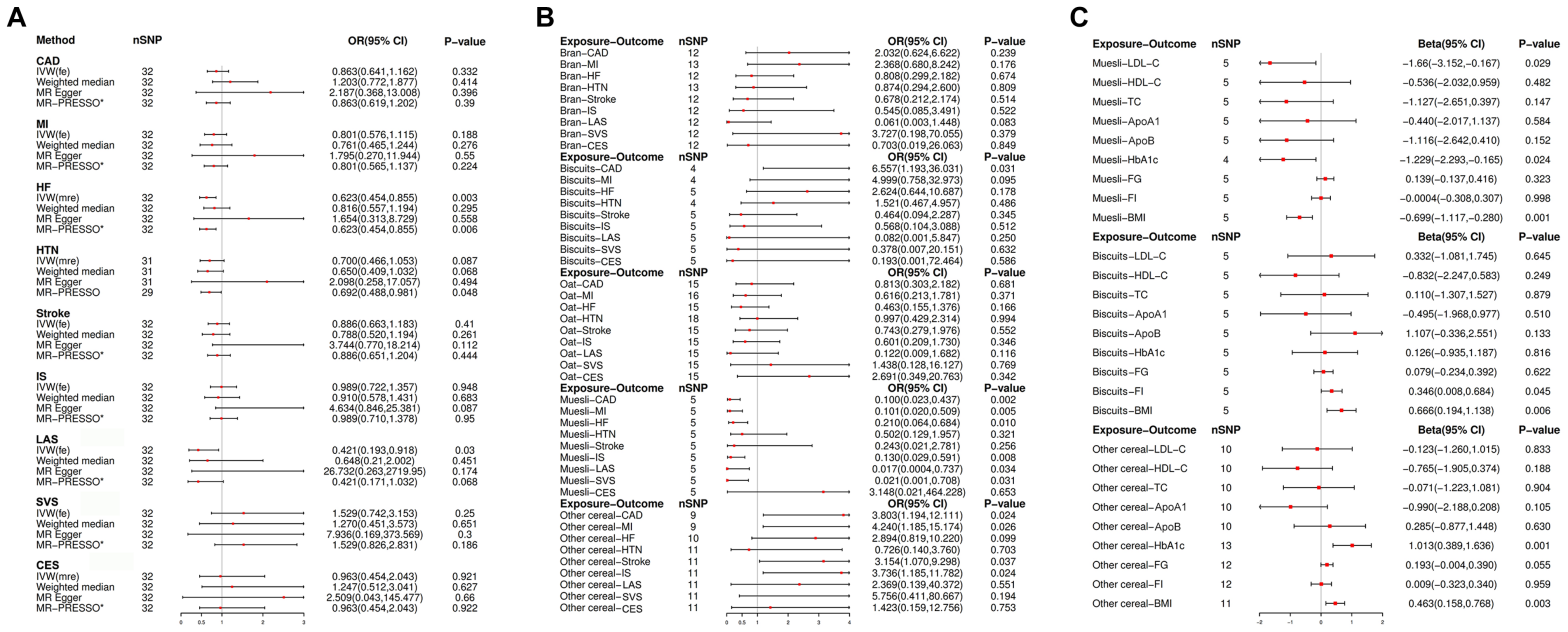
**(A)** Univariable Mendelian randomization (UVMR) estimates of the causal associations of cereal intake with CVD. **(B)** UVMR estimates of the causal associations of cereal type with CVD using the inverse-variance weighting (IVW) method. **(C)** UVMR estimates of the causal associations of cereal type with the mediator using the IVW method. OR, odd ratio; CI, confidence interval; SNP, single nucleotide polymorphism; IVW (fe), fixed effects inverse-variance weighting; IVW (mre), multiplicative random IVW; MR-PRESSO, MR-pleiotropy residual sum and outlier; ^*^No outlier was detected; CHD, coronary heart disease; MI, myocardial infarction; HTN, hypertension; HF, heart failure; IS, ischemic stroke; LAS, large artery stroke; SVS, small vessel stroke; CES, cardioembolic stroke; BMI, body mass index; TC, total cholesterol; LDL-C, low-density lipoprotein total cholesterol; HDL-C, high-density lipoprotein total cholesterol; ApoA1, apolipoprotein A-I; ApoB, apolipoprotein B; HbA1c, glycosylated hemoglobin; FG, fasting glucose; FI, fasting insulin.

The results of the genetically predicted causal associations of cereal intake with different CVDs are shown in Figure 3A. The results of the IVW analyses suggested that genetically predicted cereal intake is significantly associated with the reduced risk of HF (OR: 0.623; 95% CI [0.454, 0.85]; *p* = 0.003) and suggestively associated with the reduced risk of LAS (OR: 0.421; 95% CI [0.193, 0.918]; *p* = 0.030). However, there is insufficient evidence to suggest that genetically predicted cereal intake has a causal relationship with other CVDs.

Figure 3B and Supplementary Tables S7–S11 show the genetically predicted causal effect between various types of cereal as the primary cereal intake and CVDs. The results of the IVW analyses suggested that genetically predicted biscuits as the primary cereal intake is suggestively associated with an increased risk of CHD (OR: 6.557; 95% CI [1.197, 36.031]; *p* = 0.031); and genetically predicted other cereals as the primary cereal intake is suggestively associated with the risk of CHD (OR: 3.803; 95% CI [1.194, 12.111]; *p* = 0.024), MI (OR: 4.240; 95% CI [1.185, 15.174]; *p* = 0.026), stroke (OR: 3.154; 95% CI [1.070, 9.298]; *p* = 0.037), and IS (OR: 3.736; 95% CI [1.185, 11.782]; *p* = 0.024). However, muesli as the primary cereal intake is significantly associated with a reduced risk of CHD (OR: 0.100; 95% CI [0.023, 0.437]; *p* = 0.002), MI (OR: 0.101; 95% CI [0.020, 0.509]; *p* = 0.005), and SVS (OR: 0.021; 95% CI [0.001, 0.708]; *p* = 0.005), and suggestively associated with the risk of HF (OR: 0.210; 95% CI [0.064, 0.684]; *p* = 0.010), IS (OR: 0.130; 95% CI [0.029, 0.591]; *p* = 0.008), and LAS (OR: 0.017; 95% CI [0.0004, 0.737]; *p* = 0.034).

In Figure 3C and Supplementary Tables S12–S14, the correlation between genetically predicted muesli, biscuits, and other cereals as primary cereal intake and cardiovascular risk factors is presented. IVW analyses revealed that genetically predicted muesli as the primary cereal intake was significantly associated with a reduction in BMI (beta: −0.699; 95% CI [−1.117, −0.280]; *p* = 0.001) and suggestively associated with a reduction in LDL-C levels (beta: −1.660; 95% CI [−3.152, −0.167]; *p* = 0.029) and HbA1c levels (beta: −1.229; 95% CI [−2.293, −0.165]; *p* = 0.024). Conversely, cereals of a different type as the primary cereal intake were found to significantly increase the levels of BMI (beta: 0.463; 95% CI [0.158, 0.768]; *p* = 0.003) and HbA1c (beta: 1.013; 95% CI [0.389, 1.636]; *p* = 0.001); biscuits as the primary cereal intake were suggestively associated with an increase in BMI (beta: 0.666; 95% CI [0.194, 1.138]; *p* = 0.006) and FI (beta: 0.346; 95% CI [0.008, 0.684]; *p* = 0.045).

Most of the statistical models showed directional estimates consistent with the IVW analysis, as depicted in Figure 3 and Supplementary Tables S7–S14. In the assessment of causal effects using Cochran’s Q test, we noted heterogeneity among the employed SNPs (Supplementary Table 15). We used the multiplicative random effects IVW approach for these MR analyses, while employing the fixed-effects model for other MR analyses. Furthermore, there was no evidence of deviation from zero in the intercepts of the MR-Egger analysis, but we found that the MR-PRESSO global test *p* = 0.015 in the MR analyses of cereal intake on HF indicated the existence of horizontal pleiotropy (Supplementary Table S15). In addition, scatter plots illustrated the relationships between cereal intake and type on several CVD risks (Supplementary Figures S1–S6).

### 3.3 Causal estimates of genetic susceptibility to each mediator of CVD adjusted for cereal type

To explore the mediating effect, we evaluated the effect of potential mediators in the causal relationships that showed significance. In the MVMR results, each 1-SD unit with higher BMI was significantly associated with the risk of CHD (IVW OR: 1.480; 95% CI [1.309–1.673]), MI (IVW OR: 1.430; [95% CI: 1.266–1.616]), and HF (IVW OR: 1.589; 95% CI [1.447–1.746]) after adjusting for muesli; each 1-SD unit with higher LDL-C was significantly associated with the risk of CHD (IVW OR: 1.409; 95% CI [1.279–1.552]), MI (IVW OR: 1.374; 95% CI [1.240–1.522]), IS (IVW OR: 1.106; 95% CI [1.047–1.167]), and LAS (IVW OR: 1.263; 95% CI [1.117–1.428]), and suggestively associated with the risk of HF (IVW OR: 1.076; 95% CI [1.005–1.152]) after adjusting for muesli; each 1-SD unit with higher HbA1c was significantly associated with the risk of CHD (IVW OR: 1.165; 95% CI [1.087–1.247]) and MI (IVW OR: 1.154; 95% CI [1.073–1.241]) and suggestively associated with the risk of LAS (IVW OR: 1.234; 95% CI [1.044–1.458]) after adjusting for muesli (Table 2).

**Table 2:** The multivariable Mendelian randomization results of mediators on cardiovascular diseases adjusted for cereal type.

Mediator	Outcome	Number of SNPs	OR (95%CI)	Beta (95%CI)	p
Mediators on cardiovascular diseases adjusted for muesli as the primary cereal intake					
BMI	Coronary heart disease	151	1.480 (1.309, 1.673)	0.392 (0.269, 0.515)	3.740 × 10^−10^
BMI	Myocardial infarction	151	1.430 (1.266, 1.616)	0.358 (0.236, 0.480)	9.341 × 10^−09^
BMI	Heart failure	151	1.589 (1.447, 1.746)	0.463 (0.369, 0.557)	4.486 × 10^−22^
BMI	Large artery stroke	151	1.135(0.883, 1.458)	0.127 (0.124, 0.377)	0.322
BMI	Small vessel stroke	151	0.861 (0.699, 1.059)	−0.150(−0.358, 0.057)	0.156
BMI	Ischemic stroke	151	1.059 (0.948, 1.183)	0.057(−0.054, 0.168)	0.311
LDL-C	Coronary heart disease	33	1.409 (1.279, 1.552)	0.343 (0.246, 0.439)	3.883 × 10^−12^
LDL-C	Myocardial infarction	33	1.374 (1.240, 1.522)	0.318 (0.215, 0.420)	1.188 × 10^−09^
LDL-C	Heart failure	33	1.076 (1.005, 1.152)	0.0733 (0.005, 0.141)	0.034
LDL-C	Large artery stroke	33	1.263 (1.117, 1.428)	0.233 (0.111, 0.356)	0.0002
LDL-C	Small vessel stroke	33	1.079 (0.971, 1.200)	0.076(−0.030, 0.182)	0.159
LDL-C	Ischemic stroke	33	1.106 (1.047, 1.167)	0.100 (0.046, 0.155)	0.0003
HbA1c	Coronary heart disease	44	1.165 (1.087, 1.247)	0.152 (0.084, 0.221)	1.324 × 10^−05^
HbA1c	Myocardial infarction	44	1.154 (1.073, 1.241)	0.143 (0.070, 0.216)	0.0001
HbA1c	Heart failure	44	0.998 (0.942, 1.056)	−0.002 (−0.059, 0.055)	0.932
HbA1c	Large artery stroke	44	1.234 (1.044, 1.458)	0.210 (0.043, 0.377)	0.014
HbA1c	Small vessel stroke	44	1.104 (0.968, 1.259)	0.099 (−0.033, 0.230)	0.142
HbA1c	Ischemic stroke	44	1.016 (0.950, 1.086)	0.016 (−0.051, 0.082)	0.644
Mediators on cardiovascular diseases adjusted for other cereals as the primary cereal intake					
HbA1c	Coronary heart disease	43	1.173 (1.065, 1.291)	0.160 (0.063, 0.256)	0.001
HbA1c	Myocardial infarction	43	1.161 (1.050, 1.283)	0.149 (0.049, 0.249)	0.004
HbA1c	Total stroke	43	1.016 (0.942, 1.095)	0.016(−0.060, 0.091)	0.686
HbA1c	Ischemic stroke	43	1.025 (0.948, 1.107)	0.024(−0.053, 0.102)	0.537
BMI	Coronary heart disease	149	1.570 (1.388, 1.776)	0.451 (0.328, 0.574)	7.664 × 10^−13^
BMI	Myocardial infarction	149	1.547 (1.368, 1.750)	0.437 (0.313, 0.560)	3.804 × 10^−12^
BMI	Total stroke	151	1.167 (1.051, 1.295)	0.154 (0.050, 0.258)	0.004
BMI	Ischemic stroke	151	1.176 (1.047, 1.321)	0.162 (0.046, 0.278)	0.006
Mediators on cardiovascular diseases adjusted for biscuit cereal as the primary cereal intake					
Fasting insulin	Coronary heart disease	37	1.161 (1.081, 1.246)	0.149 (0.078, 0.220)	3.870 × 10^−05^
BMI	Coronary heart disease	147	1.553 (1.377, 1.752)	0.440 (0.320, 0.561)	7.002 × 10^−13^

MVMR, multivariable Mendelian randomization; nSNP, the number of single nucleotide polymorphisms; OR, odds ratio; CI, confidence interval; IVW, inverse-variance weighting; BMI, body mass index; LDL-C, low-density lipoprotein total cholesterol; HbA1c, glycosylated hemoglobin.

Moreover, each 1-SD unit with higher HbA1c increased the risk of CHD (OR: 1.173; 95% CI [1.065–1.291]) and MI (OR: 1.161; 95% CI [1.050–1.283]) after adjustment for other cereals; each 1-SD unit with higher BMI increased the risk of CHD (OR: 1.570; 95% CI [1.388–1.776]), MI (OR: 1.547; 95% CI [1.368–1.750]), stroke (OR: 1.167; 95% CI [1.051–1.295]), IS (OR: 1.176; 95% CI [1.047–1.321]) after adjustment for other cereals (Table 2).

Also, each 1-SD unit with higher fasting insulin (OR: 1.161; 95% CI [1.081–1.246]) and BMI (OR: 1.553; 95% CI [1.377–1.752]) was significantly associated with an increased risk of CHD after adjustment for biscuits (Table 2).

Finally, the mediating effects of the mediators in the association between cereal type and CVD were assessed, including BMI (muesli on CHD: proportion mediated = 11.89%; muesli on MI: proportion mediated = 10.90%; muesli on HF: proportion mediated = 20.72%; other cereal on CHD: proportion mediated = 15.64%; other cereal on MI: proportion mediated = 14.00%; other cereal on stroke: proportion mediated = 6.21%; other cereal on IS: proportion mediated = 5.70%; biscuit cereal on CHD: proportion mediated = 15.59%), LDL-C (muesli on CHD: proportion mediated = 24.68%; muesli on MI: proportion mediated = 22.99%; muesli on HF: proportion mediated = 7.79%; muesli on LAS: proportion mediated = 9.47%; muesli on IS: proportion mediated = 8.17%;), HbA1c (muesli on CHD: proportion mediated = 8.13%; muesli on MI: proportion mediated = 7.66%; muesli on LAS: proportion mediated = 6.33%; other cereal on CHD: proportion mediated = 12.10%; other cereal on MI: proportion mediated = 10.47%) and fasting insulin (biscuit cereal on CHD: proportion mediated = 11.91%) (Table 3).

**Table 3:** Mendelian randomization estimates of proportions mediated by mediators in the causal association between cereal type and cardiovascular disease.

Mediator	Total effect	Direct effect	Indirect effect	Proportion mediated %
BMI				
Muesli on CHD	−2.304	−2.030	−0.274	11.89%
Muesli on MI	−2.294	−2.044	−0.250	10.90%
Muesli on HF	−1.562	−1.239	−0.324	20.72%
Other cereal on CHD	1.336	1.127	0.209	15.64%
Other cereal on MI	1.444	1.242	0.202	14.00%
Other cereal on stroke	1.149	1.077	0.071	6.21%
Other cereal on IS	1.318	1.243	0.075	5.70%
Biscuit cereal on CHD	1.881	1.587	0.293	15.59%
LDL-C				
Muesli on CHD	−2.304	−1.735	−0.569	24.68%
Muesli on MI	−2.294	−1.767	−0.527	22.99%
Muesli on HF	−1.562	−1.441	−0.122	7.79%
Muesli on LAS	−4.086	−3.699	−0.387	9.47%
Muesli on IS	−2.040	−1.873	−0.167	8.17%
HbA1c				
Muesli on CHD	−2.304	−2.117	−0.187	8.13%
Muesli on MI	−2.294	−2.118	−0.176	7.66%
Muesli on LAS	−4.086	−3.828	−0.258	6.33%
Other cereal on CHD	1.336	1.174	0.162	12.10%
Other cereal on MI	1.444	1.293	0.151	10.47%
Fasting insulin				
Biscuit cereal on CHD	1.881	1.656	0.224	11.91%

Total effect = β1; Indirect effect = β2*β3; Direct effect = β1–β2*β3; Proportion mediated = (β2*β3)/β1. CHD, coronary heart disease; MI, myocardial infarction; HF, heart failure; IS, ischemic stroke; LAS, large artery stroke; BMI, body mass index; LDL-C, low-density lipoprotein total cholesterol; HbA1c, glycosylated hemoglobin.

### 3.4 Causal estimates of genetic susceptibility of cereal intake and cereal type to CVD adjusted for SES

SES often affects dietary habits, and individuals who have a habit of consuming muesli may have higher SES, so we used MVMR to assess the causality of cereal intake and type on CVD after adjusting for SES, including education and income. We found that genetically predicted muesli as the primary cereal intake was significantly associated with the reduced risk of HF (OR: 0.210; 95% CI [0.088, 0.498]; *p* = 3.93 × 10^−04^); after adjusting for education, genetically predicted muesli as the primary cereal intake was suggestively associated with a reduced risk of LAS (OR: 0.020; 95% CI [0.001, 0.762]; *p* = 0.035). However, there is no evidence proving the causal relationship between cereal type and other CVDs after adjusting for education or income (Table 4).

**Table 4:** The multivariable Mendelian randomization estimating the associations of cereal type with the mediators after adjusting for education or income by the IVW method.

Exposure	Outcome	After adjusting for education		After adjusting for income	
		IVW OR (95% CI)	*p* value	IVW OR (95% CI)	*p* value
Cereal intake	LAS	0.510 (0.257,1.014)	0.055	0.494 (0.228,1.071)	0.074
Muesli	IS	0.516 (0.211,1.259)	0.146	0.413 (0.098,1.735)	0.227
Muesli	LAS	0.118 (0.013,1.055)	0.056	1.113 (0.032,39.216)	0.953
Muesli	SVS	0.644 (0.093,4.474)	0.657	0.020 (0.001,0.762)	0.035
Muesli	HF	0.210 (0.088,0.498)	3.93 × 10^−04^	0.308 (0.084,1.132)	0.076
Muesli	MI	0.617 (0.225,1.695)	0.349	0.184 (0.029,1.151)	0.070
Biscuit cereal	CHD	0.676 (0.275,1.658)	0.392	0.225 (0.040,1.250)	0.088
Other cereal	CHD	0.804 (0.261,2.471)	0.703	4.948 (0.38,64.394)	0.222
Other cereal	CHD	0.893 (0.361,2.207)	0.806	0.183 (0.028,1.206)	0.077
Other cereal	MI	1.054 (0.382,2.908)	0.919	0.174 (0.024,1.286)	0.087
Other cereal	Stroke	1.354 (0.604,3.037)	0.462	1.485 (0.367,6.015)	0.579
Other cereal	IS	1.439 (0.589,3.517)	0.425	1.548 (0.361,6.633)	0.556

IVW, inverse-variance weighting; IS, ischemic stroke; LAS, large artery stroke; SVS, small vessel stroke; HF, heart failure; CHD, coronary heart disease; MI, myocardial infarction.

## 4 Discussion

Several observational studies have indicated a correlation between cereal consumption and CVD risk (19). Through a series of MR analyses, we have systematically established the potential causality between genetically predicted cereal intake and a reduced LAS risk, in addition to the genetic liability of muesli as the primary cereal intake to be causally associated with a reduced risk of several CVDs and cardiovascular metabolic factors, including CHD, MI, HF, LAS, SVS, BMI, LDL-C, and HbA1c. Conversely, biscuits and other cereals, such as cornflakes, as the primary cereal intake are associated with an increased risk of CVDs and cardiovascular metabolic factors, such as HbA1c, fasting insulin, and BMI. It is noteworthy that, after adjusting for SES, most causal relationships are no longer statistically significant.

Recent prospective cohort studies found that higher consumption of whole-grain cold breakfast cereals and bran was associated with a reduced risk of IS (20). However, a meta-analysis showed that intake levels of whole grains were non-significantly associated with stroke risk (19). These results align with our findings. Although we did not find a causal relationship between cereal intake and total stroke and IS, the findings of our MR analysis suggest that genetically determined cereal consumption may lower the risk of LAS. In addition, we found an causal association between cereal intake and HF, which was largely driven by horizontal pleiotropy (MR-PRESSO global test *p* = 0.015).

Subsequently, we conducted an MR analysis in populations with a habit of consuming cereal and found that muesli as the primary cereal intake was negatively correlated with various CVDs and their risk factors. In contrast, biscuit cereal and other cereals, such as cornflakes, were positively correlated with CVDs and their risk factors. A population-based longitudinal study reported that muesli was significantly protective against CVDs, stroke, and diabetes (8). Moreover, an Australian longitudinal study showed that although consumption of higher-fiber (whole-grain) cereals did not protect against diabetes (10), muesli was associated with a reduction in obesity (7) and diabetes (10). Obesity and diabetes are widely known to be the most common CVD risk factors. We found consistent results that genetically predicted muesli reduced the risk of CHD, MI, HF, IS, LAS, and SVS, in addition to the levels of BMI, LDL-C, and HbA1c. Although our MR analysis did not reveal a significant association between muesli consumption and diabetes, HbA1c serves as a marker of blood glucose levels over the previous 2–3 months and is commonly utilized to assess glycemic control in diabetic patients. A value ≥6.5% is one of the diagnostic criteria for diabetes.

Breakfast muesli often contains nuts and dried fruits. Epidemiological studies have demonstrated that dried fruits and nuts provide protection from CVDs (21–23). Nuts and dried fruits are rich sources of several nutrients and important bioactive compounds, such as dietary fiber, polyphenols, minerals, vitamins, and antioxidants. These components may have a significant effect on modulating CVD and diabetes risk (21, 22, 24, 25). A recent MR study suggested that dried fruit intake reduces the risk of HF, total IS, and SVS, which further validates our findings (26).

Reduction of LDL-C levels and glycemic control are primary goals for CVD prevention. Consumption of dietary fiber can reduce insulin response, blood cholesterol, and glucose levels (27). An increasing number of studies have shown that the consumption of β-glucan from oats and barley may be associated with a reduced risk of CVD (28–30). Another meta-analysis showed that oat-bran-enriched diets containing oat β-glucan reduced LDL and total cholesterol (31). Whole-grain cereals are rich in essential fatty acids, protein, dietary fiber, and calcium, which have been shown to reduce LDL-C (32).

Nuts and dried fruits also have similar protective effects. A systematic review showed that tree nut intake reduced TC, LDL-C, apolipoprotein B, and triglycerides but had no effect on HDL-C (33). Another earlier analysis of pooled individual data from 21 randomized controlled studies also indicated that consumption of tree nuts or peanuts reduced LDL-C (34). In addition, a meta-analysis showed that consumption of tree nuts or peanuts significantly reduced fasting insulin (35). However, the relationship between dried fruit intake and LDL-C is conflicting, and different types of dried fruits may yield contradictory results (36–39). Furthermore, current research findings suggest that dried fruits are beneficial for postprandial blood glucose regulation and glycemic control in patients with diabetes (25). The effects of nuts, dried fruits, and cereal on LDL-C and glycemic control may explain the partial mechanism by which muesli reduces CVD. The relationship warrants further investigation.

A cross-sectional study from The Gambia showed that the consumption of snacks, such as biscuits, was significantly associated with obesity and overweight (40). Another study from the British Regional Heart Study reported that a high-sugar dietary pattern, including a diet high in biscuits, was associated with a borderline significant trend for an increased risk of CHD events (41). These results align with our findings. Our MR analysis confirms the causal relationship between biscuits, CHD, and BMI. We speculate that the negative role of biscuits on CVD may be caused by their ingredients, trans fatty acids, saturated fatty acids, and acrylamide (42–44), which have been reportedly involved in CVD and diabetes (45–48). Although a study of 399 participants who underwent coronary angiography showed that consuming unsweetened cornflakes had a protective effect against CVD (49), our results indicate that other cereals, such as cornflakes, increase the risk of CVD and HbA1c levels. Cornflakes often contain high amounts of sugar and other additives during processing. Long-term intake of foods high in sugar can lead to an increase in HbA1c levels. High blood glucose is an important risk factor for CVD. In addition, a higher intake of regular popcorn has been linked to an elevated risk of IS and CHD (20, 50). During food processing, sodium, butter, and trans fat are often added to regular popcorn, which may at least partially explain this positive correlation.

In addition to cardiovascular metabolic factors, SES is also known to be an important factor affecting CVD, and individuals of different SES often have different dietary habits. Several studies have reported that individuals with moderate and high SES consume more cereal compared to those with low SES (51, 52) and that women with higher SES consume more muesli (53). Our results indicated that the most significant effect of cereal consumption on CVD disappeared after adjustment for education or income, implying that SES may play a more important role than cereal consumption in most CVD risks.

Inevitably, certain limitations of our study should be acknowledged. First, it is important to note that all participants involved in the study were of European ethnicity. It is well known that the risk factors, prevalence, and mortality of CVD vary among ethnic groups. Therefore, it is possible that our findings may not explain the potential causality of cereal intake and cereal type on CVD in other ethnicities and populations. Second, due to the use of data from the UK Biobank, the population of the GWAS of cereal type and BMI may have partially overlapped. Third, the MR analysis of the association between cereal types and CVD is limited to datasets of populations with cereal consumption habits and cannot assess the causal relationship between specific cereal types and CVD compared to non-cereal consumers. Dietary data collected through questionnaires may cause recall bias, and some participants may consume multiple cereal types simultaneously, with no significant gap in consumption between different cereal types. Using the main cereal types consumed as the exposure of this population may not effectively represent their dietary habits and thus may not produce robust results. Fourth, the partial MR analyses indicated only suggestive causal relationships after correction for multiple testing. Further randomized controlled studies are warranted to clarify causal relationships. Finally, muesli is a healthy dietary habit. People with cereal consumption habits may have other healthy diets, such as vegetables, fruits, and low-calorie diets. These unmeasured confounders may influence our findings.

## 5 Conclusion

The results of our study underscore the causal beneficial influence of muesli as the primary cereal intake on CVD. Our findings suggest that an adequate intake of muesli may be beneficial for the primary prevention of CVDs.

## Data availability statement

The original contributions presented in the study are included in the article/Supplementary material, further inquiries can be directed to the corresponding author.

## Author contributions

JL: Conceptualization, Data curation, Formal analysis, Methodology, Validation, Writing – original draft. DC: Conceptualization, Supervision, Validation, Writing – review & editing.
