# Supplementary material for: Causal relationship of cereal intake and type with cardiovascular disease: a Mendelian randomization study
Source: Front Nutr. 2024 Jan 23;10:1320120. doi: 10.3389/fnut.2023.1320120 (PMC10844472; doi:10.3389/fnut.2023.1320120)
Supplement: Supplementary file 1 [file Table_1.DOCX]

Supplementary Material

Supplementary Table 1 Characteristics of SNPs used as instrumental variables for cereal intake.

Supplementary Table 2 Characteristics of SNPs used as instrumental variables for bran cereal.

Supplementary Table 3 Characteristics of SNPs used as instrumental variables for biscuit cereal.

Supplementary Table 4 Characteristics of SNPs used as instrumental variables for oat cereal.

Supplementary Table 5 Characteristics of SNPs used as instrumental variables for muesli.

Supplementary Table 6 Characteristics of SNPs used as instrumental variables for other cereal.

Supplementary Table 7 MR estimating the associations of bran cereal with cardiovascular diseases.

Supplementary Table 8 MR estimating the associations of biscuit cereal with cardiovascular diseases.

Supplementary Table 9 MR estimating the associations of oat cereal with cardiovascular diseases.

Supplementary Table 10 MR estimating the associations of muesli with cardiovascular diseases.

Supplementary Table 11 MR estimating the associations of other cereal with cardiovascular diseases.

Supplementary Table 12 MR estimating the associations of biscuit cereal with mediator.

Supplementary Table 13 MR estimating the associations of muesli with mediator.

Supplementary Table 14 MR estimating the associations of other cereal with mediator.

Supplementary Table 15 Heterogeneity and pleiotropy tests for the associations between cereal intake and type and cardiovascular diseases.

Supplementary Figure 1 Scatter plot using all IVs of cereal intake on CVDs.

Supplementary Figure 2 Scatter plot using all IVs of bran cereal on CVDs.

Supplementary Figure 3 Scatter plot using all IVs of biscuit cereal1 on CVDs.

Supplementary Figure 4 Scatter plot using all IVs of oat cereal on CVDs.

Supplementary Figure 5 Scatter plot using all IVs of muesli on CVDs.

Supplementary Figure 6 Scatter plot using all IVs of other cereal on CVDs.

Supplementary Figure 7 Scatter plot using all IVs of biscuit cereal on CVD risk factors.

Supplementary Figure 8 Scatter plot using all IVs of muesli on CVD risk factors.

Supplementary Figure 9 Scatter plot using all IVs of other cereal on CVD risk factors.

Abbreviation: SNP, Single nucleotide polymorphism; MR, Mendelian randomization; CVD, cardiovascular disease; IVs, instrumental variables; CHD,coronary heart disease; MI, myocardial infarction; HTN, hypertension; HF, heart failure; IS, ischemic stroke; ISla, ischemic stroke (large artery atherosclerosis); ISsv, ischemic stroke (small-vessel); ISce, ischemic stroke (cardioembolic); BMI, body mass index; TC, total cholesterol; LDL-C, total cholesterol in low density lipoprotein; HDL-C, total cholesterol in high density lipoprotein;apA-I, Apolipoprotein A-I; apB, Apolipoprotein B; HbA1c, glycosylated hemoglobin; FG, Fasting glucose; FI, Fasting insulin.

MR, Mendelian randomization; A1= effect_allele; A2= other_allele.

EAF, Effect allele frequency; SE, Standard error.

R^2^=2×EAF×(1-EAF)×beta^2/(2×EAF×(1-EAF)×beta^2)+2×EAF×(1-EAF)×se×N×beta^2). F-statistics= (n-k-1)R^2^/k(1-R^2^).

(N = the sample size, k= the number of used SNPs, and R^2^ = the cumulative explained variance of the used SNPs)

Supplementary Table 1 Characteristics of SNPs used as instrumental variables for cereal intake.

|  | SNP | chr | pos | effect_  allele | other_  allele | EAF | Beta | SE | pval | F-statistic |
| --- | --- | --- | --- | --- | --- | --- | --- | --- | --- | --- |
| 1 | rs12354267 | 1 | 44248272 | C | T | 0.31 | 0.012 | 0.002 | 1.70E-09 | 17.25 |
| 2 | rs112780312 | 1 | 153797015 | A | G | 0.27 | -0.012 | 0.002 | 1.80E-09 | 16.51 |
| 3 | rs184643 | 2 | 45189441 | A | G | 0.57 | -0.012 | 0.002 | 1.60E-11 | 18.47 |
| 4 | rs6545770 | 2 | 60204427 | T | A | 0.75 | -0.014 | 0.002 | 2.70E-11 | 16.18 |
| 5 | rs67723420 | 3 | 35775115 | A | T | 0.38 | 0.011 | 0.002 | 1.20E-08 | 18.05 |
| 6 | rs7619139 | 3 | 25110415 | A | T | 0.59 | -0.017 | 0.002 | 9.70E-21 | 18.36 |
| 7 | rs11097340 | 4 | 93465297 | T | C | 0.40 | -0.012 | 0.002 | 2.10E-10 | 18.36 |
| 8 | rs3115230 | 4 | 123009766 | A | C | 0.75 | -0.011 | 0.002 | 3.00E-08 | 16.09 |
| 9 | rs10057775 | 5 | 87469663 | C | T | 0.89 | 0.020 | 0.003 | 4.50E-12 | 11.52 |
| 10 | rs79642906 | 5 | 59650336 | A | G | 0.08 | -0.018 | 0.003 | 1.90E-08 | 10.33 |
| 11 | rs1853931 | 6 | 125170409 | A | G | 0.53 | -0.011 | 0.002 | 3.80E-10 | 18.41 |
| 12 | rs6918737 | 6 | 19721681 | A | T | 0.23 | 0.014 | 0.002 | 7.60E-11 | 15.80 |
| 13 | rs2817377 | 6 | 50789176 | A | G | 0.54 | 0.010 | 0.002 | 3.10E-08 | 18.63 |
| 14 | rs4410790 | 7 | 17284577 | C | T | 0.63 | -0.011 | 0.002 | 3.40E-09 | 18.05 |
| 15 | rs13234131 | 7 | 73025975 | G | A | 0.13 | 0.017 | 0.003 | 1.60E-10 | 12.53 |
| 16 | rs4739095 | 8 | 64391730 | A | G | 0.77 | -0.013 | 0.002 | 9.90E-10 | 15.83 |
| 17 | rs2927238 | 8 | 76758514 | G | T | 0.61 | 0.010 | 0.002 | 2.10E-08 | 18.23 |
| 18 | rs2799849 | 9 | 86752641 | T | C | 0.68 | -0.012 | 0.002 | 9.80E-11 | 17.49 |
| 19 | rs7040561 | 9 | 128528978 | A | T | 0.85 | -0.016 | 0.003 | 1.10E-10 | 13.23 |
| 20 | rs2450126 | 11 | 77916279 | G | A | 0.16 | -0.015 | 0.002 | 1.30E-09 | 13.57 |
| 21 | rs10837531 | 11 | 40966859 | G | C | 0.46 | 0.011 | 0.002 | 2.00E-09 | 18.55 |
| 22 | rs11038810 | 11 | 46210773 | G | A | 0.64 | 0.011 | 0.002 | 2.30E-09 | 17.89 |
| 23 | rs627185 | 11 | 121553314 | G | C | 0.54 | -0.011 | 0.002 | 1.50E-09 | 18.61 |
| 24 | rs2472297 | 15 | 75027880 | T | C | 0.26 | -0.016 | 0.002 | 4.50E-15 | 16.49 |
| 25 | rs68136852 | 17 | 27382061 | A | C | 0.15 | -0.014 | 0.002 | 1.20E-08 | 13.45 |
| 26 | rs3859193 | 17 | 38125856 | A | T | 0.47 | -0.010 | 0.002 | 9.50E-09 | 18.52 |
| 27 | rs4797242 | 18 | 6407483 | A | C | 0.30 | 0.011 | 0.002 | 4.50E-09 | 17.10 |
| 28 | rs11670024 | 19 | 18818335 | G | A | 0.12 | 0.016 | 0.003 | 1.10E-08 | 11.91 |
| 29 | rs6510177 | 19 | 31211647 | C | T | 0.81 | -0.013 | 0.002 | 1.20E-08 | 14.57 |
| 30 | rs78854891 | 19 | 42752078 | C | T | 0.07 | 0.022 | 0.004 | 1.10E-09 | 9.17 |

Supplementary Table 2 Characteristics of SNPs used as instrumental variables for bran cereal.

|  | SNP | chr | pos | effect_  allele | other_  allele | EAF | Beta | SE | pval | F-statistic |
| --- | --- | --- | --- | --- | --- | --- | --- | --- | --- | --- |
| 1 | rs3129559 | 1 | 246814137 | A | G | 0.56 | 0.005 | 0.001 | 3.11E-06 | 73.13 |
| 2 | rs147186763 | 2 | 207843967 | A | C | 0.27 | -0.005 | 0.001 | 4.24E-06 | 63.90 |
| 3 | rs144959869 | 2 | 77297972 | C | G | 0.02 | -0.018 | 0.004 | 2.68E-06 | 19.01 |
| 4 | rs17236659 | 6 | 100243033 | G | A | 0.21 | 0.005 | 0.001 | 4.58E-06 | 60.36 |
| 5 | rs9322203 | 6 | 149955366 | G | A | 0.32 | 0.005 | 0.001 | 2.80E-06 | 68.16 |
| 6 | rs6592945 | 7 | 51719529 | A | T | 0.52 | -0.005 | 0.001 | 2.56E-06 | 73.49 |
| 7 | rs10225302 | 7 | 31001075 | T | C | 0.41 | 0.005 | 0.001 | 1.11E-07 | 71.52 |
| 8 | rs11562791 | 8 | 119265007 | T | C | 0.01 | 0.030 | 0.006 | 2.05E-06 | 11.21 |
| 9 | rs13274406 | 8 | 31459960 | C | T | 0.28 | -0.005 | 0.001 | 1.10E-06 | 65.57 |
| 10 | rs145549010 | 8 | 125865260 | A | G | 0.02 | 0.019 | 0.004 | 1.44E-06 | 17.84 |
| 11 | rs11047165 | 12 | 24071751 | C | T | 0.29 | 0.005 | 0.001 | 1.38E-06 | 66.87 |
| 12 | rs548159361 | 14 | 63344922 | C | G | 0.02 | 0.016 | 0.003 | 3.28E-06 | 21.32 |
| 13 | rs79741827 | 16 | 56733116 | C | T | 0.04 | -0.011 | 0.002 | 4.65E-06 | 30.21 |
| 14 | rs6056882 | 20 | 9774742 | A | G | 0.03 | 0.013 | 0.003 | 4.32E-06 | 26.12 |

Supplementary Table 3 Characteristics of SNPs used as instrumental variables for biscuit cereal.

|  | SNP | chr | pos | effect_  allele | other_  allele | EAF | Beta | SE | pval | F-statistic |
| --- | --- | --- | --- | --- | --- | --- | --- | --- | --- | --- |
| 1 | rs56912210 | 1 | 19974092 | G | A | 0.13 | -0.008 | 0.002 | 2.27E-07 | 130.68 |
| 2 | rs2129622 | 6 | 98595713 | A | T | 0.12 | 0.008 | 0.002 | 4.69E-08 | 130.76 |
| 3 | rs12416002 | 10 | 6793950 | T | A | 0.56 | 0.005 | 0.001 | 4.43E-07 | 200.64 |
| 4 | rs11160772 | 14 | 104363528 | A | G | 0.43 | 0.006 | 0.001 | 3.88E-09 | 199.34 |
| 5 | rs189381586 | 22 | 41314428 | A | C | 0.01 | -0.025 | 0.005 | 4.37E-07 | 39.81 |

Supplementary Table 4 Characteristics of SNPs used as instrumental variables for oat cereal.

|  | SNP | chr | pos | effect_  allele | other_  allele | EAF | Beta | SE | pval | F-statistic |
| --- | --- | --- | --- | --- | --- | --- | --- | --- | --- | --- |
| 1 | rs9426949 | 1 | 157300992 | T | C | 0.98 | -0.019 | 0.004 | 4.11E-06 | 9.70 |
| 3 | rs62153980 | 2 | 107103621 | C | G | 0.72 | -0.006 | 0.001 | 5.37E-07 | 31.97 |
| 4 | rs7597555 | 2 | 30082530 | A | G | 0.16 | -0.008 | 0.002 | 8.38E-07 | 26.19 |
| 5 | rs17400875 | 2 | 60295736 | G | T | 0.27 | -0.006 | 0.001 | 2.03E-06 | 31.52 |
| 6 | rs369232907 | 3 | 98756371 | T | G | 0.03 | -0.017 | 0.004 | 2.41E-06 | 11.28 |
| 7 | rs76987077 | 3 | 78547955 | C | T | 0.09 | 0.009 | 0.002 | 1.78E-06 | 20.29 |
| 8 | rs35386450 | 3 | 70356143 | G | T | 0.39 | 0.006 | 0.001 | 3.01E-06 | 33.28 |
| 9 | rs199693275 | 5 | 132011573 | G | A | 0.13 | 0.008 | 0.002 | 3.49E-06 | 23.16 |
| 10 | rs2273558 | 6 | 26466035 | C | A | 0.31 | -0.006 | 0.001 | 1.50E-06 | 33.16 |
| 11 | rs9272526 | 6 | 32606569 | A | G | 0.33 | -0.006 | 0.001 | 3.64E-07 | 31.66 |
| 12 | rs548439697 | 9 | 18794409 | C | A | 0.12 | -0.009 | 0.002 | 3.23E-06 | 21.80 |
| 13 | rs12378622 | 9 | 83521673 | T | C | 0.08 | 0.010 | 0.002 | 3.91E-06 | 18.84 |
| 14 | rs118097348 | 10 | 37020952 | A | T | 0.01 | 0.025 | 0.005 | 2.96E-06 | 7.55 |
| 15 | rs11000300 | 10 | 74203201 | T | C | 0.04 | 0.013 | 0.003 | 4.90E-06 | 14.39 |
| 16 | rs116896436 | 10 | 89923815 | G | A | 0.01 | 0.033 | 0.007 | 1.87E-06 | 5.78 |
| 17 | rs7936836 | 11 | 43633645 | A | C | 0.42 | 0.006 | 0.001 | 3.80E-07 | 35.21 |
| 18 | rs143623527 | 11 | 64675148 | A | G | 0.01 | -0.023 | 0.005 | 2.70E-06 | 8.31 |
| 19 | rs9538439 | 13 | 60066560 | A | G | 0.28 | 0.006 | 0.001 | 3.86E-06 | 32.16 |
| 20 | rs9529825 | 13 | 35435533 | C | G | 0.52 | -0.005 | 0.001 | 4.03E-06 | 34.86 |
| 22 | rs7203072 | 16 | 24641076 | G | A | 0.62 | -0.005 | 0.001 | 3.47E-06 | 34.58 |
| 23 | rs113502952 | 17 | 68293913 | G | C | 0.01 | -0.022 | 0.005 | 4.78E-06 | 8.39 |
| 24 | rs73027124 | 19 | 28930748 | C | T | 0.02 | 0.021 | 0.004 | 4.52E-07 | 9.75 |
| 25 | rs2770374 | 20 | 54143833 | G | T | 0.37 | 0.005 | 0.001 | 3.29E-06 | 33.91 |

Supplementary Table 5 Characteristics of SNPs used as instrumental variables for muesli.

|  | SNP | chr | pos | effect_  allele | other_  allele | EAF | Beta | SE | pval | F-statistic |
| --- | --- | --- | --- | --- | --- | --- | --- | --- | --- | --- |
| 1 | rs62174720 | 2 | 166270636 | T | C | 0.24 | 0.007 | 0.001 | 4.13E-09 | 164.37 |
| 2 | rs4852285 | 2 | 72446753 | G | A | 0.33 | 0.006 | 0.001 | 2.50E-08 | 180.87 |
| 3 | rs73093445 | 3 | 71542279 | A | C | 0.43 | 0.006 | 0.001 | 1.08E-08 | 190.36 |
| 4 | rs7748167 | 6 | 25904652 | C | A | 0.13 | -0.009 | 0.002 | 9.12E-09 | 127.84 |
| 5 | rs10995907 | 10 | 52934604 | G | C | 0.09 | 0.010 | 0.002 | 2.86E-08 | 106.22 |

Supplementary Table 6 Characteristics of SNPs used as instrumental variables for other cereal.

|  | SNP | chr | pos | effect_  allele | other_  allele | EAF | Beta | SE | pval | F-statistic |
| --- | --- | --- | --- | --- | --- | --- | --- | --- | --- | --- |
| 1 | rs61787785 | 1 | 98332523 | C | G | 0.14 | 0.007 | 0.001 | 2.77E-07 | 53.11 |
| 2 | rs17184707 | 2 | 166183577 | T | C | 0.21 | 0.006 | 0.001 | 1.81E-07 | 62.18 |
| 3 | rs57205040 | 3 | 71520890 | C | A | 0.43 | -0.006 | 0.001 | 1.13E-08 | 74.92 |
| 4 | rs142094351 | 6 | 93094870 | C | T | 0.01 | 0.025 | 0.005 | 9.84E-08 | 16.40 |
| 5 | rs9274454 | 6 | 32633371 | G | T | 0.19 | 0.012 | 0.001 | 3.68E-18 | 56.70 |
| 6 | rs3132681 | 6 | 30060041 | A | C | 0.40 | 0.005 | 0.001 | 3.55E-07 | 74.23 |
| 7 | rs1131114 | 6 | 31235869 | C | T | 0.26 | 0.007 | 0.001 | 2.95E-09 | 66.72 |
| 8 | rs6961429 | 7 | 27704400 | C | T | 0.39 | 0.005 | 0.001 | 2.11E-07 | 73.64 |
| 9 | rs6559505 | 9 | 82447233 | G | A | 0.56 | -0.005 | 0.001 | 2.96E-07 | 74.74 |
| 10 | rs11818697 | 10 | 32273277 | G | A | 0.30 | 0.006 | 0.001 | 9.74E-08 | 69.26 |
| 11 | rs2852786 | 11 | 61514085 | T | C | 0.67 | 0.006 | 0.001 | 1.74E-08 | 70.72 |
| 12 | rs2696495 | 17 | 44364864 | T | C | 0.22 | 0.008 | 0.001 | 3.98E-10 | 61.87 |
| 13 | rs624244 | 18 | 53183396 | A | G | 0.66 | 0.005 | 0.001 | 4.07E-07 | 71.07 |

Supplementary Table 7 MR estimating the associations of bran cereal with cardiovascular diseases

| Outcome | Method | No. Of  SNPs | OR (95% CI) | P value |
| --- | --- | --- | --- | --- |
| Coronary heart disease | IVW | 12 | 2.032(0.624,6.622) | 0.239 |
|  | Weighted median | 12 | 1.238(0.258,5.926) | 0.790 |
|  | MR Egger | 12 | 2.961(0.115,76.297) | 0.527 |
|  | MR PRESSO | 12 | 2.032(0.679,6.082) | 0.231 |
| Myocardial infarction | IVW | 13 | 2.368(0.680,8.242) | 0.176 |
|  | Weighted median | 13 | 1.688(0.333,8.564) | 0.528 |
|  | MR Egger | 13 | 2.441(0.135,44.279) | 0.558 |
|  | MR PRESSO | 13 | 2.368(0.752,7.455) | 0.166 |
| Heart failure | IVW | 12 | 0.808(0.299,2.182) | 0.674 |
|  | Weighted median | 12 | 1.044(0.290,3.759) | 0.948 |
|  | MR Egger | 12 | 4.609(0.293,72.471) | 0.303 |
|  | MR PRESSO | 12 | 0.808(0.333,1.958) | 0.646 |
| Hypertension | IVW | 13 | 0.874(0.294,2.600) | 0.809 |
|  | Weighted median | 13 | 0.777(0.166,3.644) | 0.749 |
|  | MR Egger | 13 | 0.108(0.007,1.706) | 0.142 |
|  | MR PRESSO | 13 | 0.874(0.320,2.392) | 0.798 |
| Stroke | IVW | 12 | 0.678(0.212,2.174) | 0.514 |
|  | Weighted median | 12 | 0.83(0.157,4.390) | 0.827 |
|  | MR Egger | 12 | 0.893(0.011,72.767) | 0.961 |
|  | MR PRESSO | 12 | 0.678(0.151,3.040) | 0.622 |
| Ischemic stroke | IVW | 12 | 0.545(0.085,3.491) | 0.522 |
|  | Weighted median | 12 | 0.654(0.110,3.887) | 0.641 |
|  | MR Egger | 12 | 1.470(0.007,314.264) | 0.891 |
|  | MR PRESSO | 11 | 1.160(0.300,4.496) | 0.834 |
| Large-artery stroke | IVW | 12 | 0.061(0.003,1.448) | 0.083 |
|  | Weighted median | 12 | 1.091(0.011,110.9) | 0.971 |
|  | MR Egger | 12 | 0.041(0.000,2850) | 0.586 |
|  | MR PRESSO | 12 | 0.061(0.001,2.620) | 0.173 |
| Small-vessel stroke | IVW | 12 | 3.727(0.198,70.055) | 0.379 |
|  | Weighted median | 12 | 1.119(0.016,79.016) | 0.959 |
|  | MR Egger | 12 | 142.458(0.021,9.78E5) | 0.297 |
|  | MR PRESSO | 12 | 3.727(0.166,83.825) | 0.425 |
| Cardioembolic stroke | IVW | 12 | 0.703(0.019,26.063) | 0.849 |
|  | Weighted median | 12 | 1.760(0.039,80.061) | 0.772 |
|  | MR Egger | 12 | 0.069(0.000,3890) | 0.642 |
|  | MR PRESSO | 11 | 2.167(0.08,58.415) | 0.655 |

Supplementary Table 8 MR estimating the associations of biscuit cereal with cardiovascular diseases

| Outcome | Method | No. of SNPs | OR (95% CI) | P value |
| --- | --- | --- | --- | --- |
| Coronary heart disease | IVW | 4 | 6.557(1.193,36.031) | 0.031 |
|  | Weighted median | 4 | 4.116(0.515,32.890) | 0.182 |
|  | MR Egger | 4 | 3.331(0.001,13500) | 0.803 |
|  | MR PRESSO | 4 | 6.557(1.922,22.368) | 0.057 |
| Myocardial infarction | IVW | 4 | 4.999(0.758,32.973) | 0.095 |
|  | Weighted median | 4 | 6.845(0.777,60.265) | 0.083 |
|  | MR Egger | 4 | 0.276(0.000,2730) | 0.810 |
|  | MR PRESSO | 4 | 4.999(1.592,15.699) | 0.070 |
| Heart failure | IVW | 4 | 2.624(0.644,10.687) | 0.178 |
|  | Weighted median | 4 | 4.311(0.667,27.857) | 0.125 |
|  | MR Egger | 4 | 0.227(0.006,8.755) | 0.485 |
|  | MR PRESSO | 4 | 2.624(0.487,14.152) | 0.325 |
| Hypertension | IVW | 5 | 1.521(0.467,4.957) | 0.486 |
|  | Weighted median | 5 | 1.339(0.298,6.023) | 0.703 |
|  | MR Egger | 5 | 2.532(0.188,34.145) | 0.534 |
|  | MR PRESSO | 5 | 1.521(0.417,5.557) | 0.560 |
| Stroke | IVW | 5 | 0.464(0.094,2.287) | 0.345 |
|  | Weighted median | 5 | 0.961(0.132,6.977) | 0.969 |
|  | MR Egger | 5 | 0.011(0.000,1.561) | 0.172 |
|  | MR PRESSO | 5 | 0.464(0.068,3.152) | 0.476 |
| Ischemic stroke | IVW | 5 | 0.568(0.104,3.088) | 0.512 |
|  | Weighted median | 5 | 1.364(0.149,12.453) | 0.784 |
|  | MR Egger | 5 | 0.001(0.000,0.156) | 0.077 |
|  | MR PRESSO | 5 | 0.568(0.050,6.484) | 0.672 |
| Large-artery stroke | IVW | 5 | 0.082(0.001,5.847) | 0.250 |
|  | Weighted median | 5 | 0.107(0.000,26.439) | 0.427 |
|  | MR Egger | 5 | 0.0002(0.000,2620) | 0.387 |
|  | MR PRESSO | 5 | 0.082(0.001,10.321) | 0.368 |
| Small-vessel stroke | IVW | 5 | 0.378(0.007,20.151) | 0.632 |
|  | Weighted median | 5 | 0.304(0.002,40.139) | 0.633 |
|  | MR Egger | 5 | 0.038(0.000,65200) | 0.685 |
|  | MR PRESSO | 5 | 0.378(0.008,18.700) | 0.651 |
| Cardioembolic stroke | IVW | 5 | 0.193(0.001,72.464) | 0.586 |
|  | Weighted median | 5 | 1.711(0.019,153.629) | 0.815 |
|  | MR Egger | 5 | 0.000(0.000,2.021) | 0.160 |
|  | MR PRESSO | 5 | 1.862(0.035,98.369) | 0.779 |

Supplementary Table 9 MR estimating the associations of oat cereal with cardiovascular diseases

| Outcome | Method | No. of SNPs | OR (95% CI) | P value |
| --- | --- | --- | --- | --- |
| Coronary heart disease | IVW | 15 | 0.813(0.303,2.182) | 0.681 |
|  | Weighted median | 15 | 0.696(0.189,2.557) | 0.585 |
|  | MR Egger | 15 | 0.819(0.045,14.941) | 0.895 |
|  | MR PRESSO | 15 | 0.813(0.277,2.388) | 0.712 |
| Myocardial infarction | IVW | 16 | 0.616(0.213,1.781) | 0.371 |
|  | Weighted median | 16 | 0.634(0.137,2.942) | 0.561 |
|  | MR Egger | 16 | 0.554(0.042,7.280) | 0.660 |
|  | MR PRESSO | 16 | 0.616(0.224,1.696) | 0.363 |
| Heart failure | IVW | 15 | 0.463(0.155,1.376) | 0.166 |
|  | Weighted median | 15 | 1.097(0.357,3.368) | 0.872 |
|  | MR Egger | 15 | 0.916(0.049,17.003) | 0.954 |
|  | MR PRESSO | 14 | 0.789(0.412,1.512) | 0.488 |
| Hypertension | IVW | 18 | 0.997(0.429,2.314) | 0.994 |
|  | Weighted median | 18 | 0.839(0.252,2.801) | 0.776 |
|  | MR Egger | 18 | 0.846(0.116,6.167) | 0.871 |
|  | MR PRESSO | 18 | 0.997(0.426,2.332) | 0.994 |
| Stroke | IVW | 15 | 0.743(0.279,1.976) | 0.552 |
|  | Weighted median | 15 | 1.364(0.314,5.933) | 0.679 |
|  | MR Egger | 15 | 4.287(0.208,88.243) | 0.363 |
|  | MR PRESSO | 15 | 0.743(0.237,2.335) | 0.619 |
| Ischemic stroke | IVW | 15 | 0.601(0.209,1.730) | 0.346 |
|  | Weighted median | 15 | 0.668(0.157,2.840) | 0.585 |
|  | MR Egger | 15 | 1.76(0.099,31.196) | 0.706 |
|  | MR PRESSO | 15 | 0.601(0.223,1.625) | 0.333 |
| Large-artery stroke | IVW | 15 | 0.122(0.009,1.682) | 0.116 |
|  | Weighted median | 15 | 0.056(0.001,2.289) | 0.128 |
|  | MR Egger | 15 | 2.229(0.001,4861.2) | 0.841 |
|  | MR PRESSO | 15 | 0.122(0.008,1.967) | 0.160 |
| Small-vessel stroke | IVW | 15 | 1.438(0.128,16.127) | 0.769 |
|  | Weighted median | 15 | 1.839(0.055,61.241) | 0.733 |
|  | MR Egger | 15 | 0.068(0.000,62.285) | 0.454 |
|  | MR PRESSO | 15 | 1.438(0.113,18.32) | 0.784 |
| Cardioembolic stroke | IVW | 15 | 2.691(0.349,20.763) | 0.342 |
|  | Weighted median | 15 | 0.751(0.042,13.343) | 0.845 |
|  | MR Egger | 15 | 4.642(0.015,1452.8) | 0.609 |
|  | MR PRESSO | 15 | 2.691(0.370,19.553) | 0.345 |

Supplementary Table 10 MR estimating the associations of muesli with cardiovascular diseases

| Outcome | Method | No. of SNPs | OR (95% CI) | P value |
| --- | --- | --- | --- | --- |
| Coronary heart disease | IVW | 5 | 0.100(0.023,0.437) | 0.002 |
|  | Weighted median | 5 | 0.138(0.017,1.122) | 0.064 |
|  | MR Egger | 5 | 1030(0.695,1.53E6) | 0.159 |
|  | MR PRESSO | 5 | 0.100(0.015,0.667) | 0.076 |
| Myocardial infarction | IVW | 5 | 0.101(0.020,0.509) | 0.005 |
|  | Weighted median |  | 0.122(0.014,1.053) | 0.056 |
|  | MR Egger | 5 | 4.147(0.001,23100) | 0.768 |
|  | MR PRESSO | 5 | 0.101(0.019,0.535) | 0.054 |
| Heart failure | IVW | 5 | 0.210(0.064,0.684) | 0.010 |
|  | Weighted median | 5 | 0.137(0.029,0.655) | 0.013 |
|  | MR Egger | 5 | 8.967(0.024,3330) | 0.520 |
|  | MR PRESSO | 5 | 0.210(0.069,0.634) | 0.050 |
| Hypertension | IVW | 5 | 0.502(0.129,1.957) | 0.321 |
|  | Weighted median | 5 | 0.379(0.056,2.562) | 0.320 |
|  | MR Egger | 5 | 472(0.633,352000) | 0.166 |
|  | MR PRESSO | 5 | 0.502(0.087,2.909) | 0.485 |
| Stroke | IVW | 5 | 0.243(0.021,2.781) | 0.256 |
|  | Weighted median | 5 | 0.201(0.026,1.572) | 0.126 |
|  | MR Egger | 5 | 0.033(0.000,45000) | 0.668 |
|  | MR PRESSO | 4 | 0.077(0.025,0.239) | 0.021 |
| Ischemic stroke | IVW | 5 | 0.13(0.029,0.591) | 0.008 |
|  | Weighted median | 5 | 0.063(0.007,0.538) | 0.011 |
|  | MR Egger | 5 | 0.004(0.000,58.028) | 0.339 |
|  | MR PRESSO | 5 | 0.130(0.022,0.770) | 0.088 |
| Large-artery stroke | IVW | 5 | 0.017(0.000,0.737) | 0.034 |
|  | Weighted median | 5 | 0.034(0.0002,5.677) | 0.195 |
|  | MR Egger | 5 | 3.54E-6(0.000,4220) | 0.324 |
|  | MR PRESSO | 5 | 0.017(0.000,0.826) | 0.109 |
| Small-vessel stroke | IVW | 5 | 0.021(0.001,0.708) | 0.031 |
|  | Weighted median | 5 | 0.014(0.000,1.008) | 0.050 |
|  | MR Egger | 5 | 7.84E-5(0.000,5200) | 0.379 |
|  | MR PRESSO | 5 | 0.021(0.005,0.094) | 0.007 |
| Cardioembolic stroke | IVW | 5 | 3.148(0.021,464.228) | 0.653 |
|  | Weighted median | 5 | 0.260(0.004,17.658) | 0.531 |
|  | MR Egger | 5 | 3.65E-5(0.000,1.9E7) | 0.512 |
|  | MR PRESSO | 5 | 3.148(0.021,464.228) | 0.676 |

Supplementary Table 11 MR estimating the associations of other cereal with cardiovascular diseases

| Outcome | Method | No. of SNPs | OR (95% CI) | P value |
| --- | --- | --- | --- | --- |
| Coronary heart disease | IVW | 9 | 3.803(1.194,12.111) | 0.024 |
|  | Weighted median | 9 | 4.809(0.895,25.846) | 0.067 |
|  | MR Egger | 9 | 0.081(0.001,10.633) | 0.346 |
|  | MR PRESSO | 9 | 3.803(0.883,16.366) | 0.111 |
| Myocardial infarction | IVW | 9 | 4.240(1.185,15.174) | 0.026 |
|  | Weighted median | 9 | 11.305(2.079,61.473) | 0.005 |
|  | MR Egger | 9 | 0.084(0.001,9.008) | 0.333 |
|  | MR PRESSO | 9 | 4.24(1.153,15.584) | 0.061 |
| Heart failure | IVW | 10 | 2.894(0.819,10.220) | 0.099 |
|  | Weighted median | 10 | 4.005(1.076,14.902) | 0.038 |
|  | MR Egger | 10 | 43.463(0.597,3163) | 0.123 |
|  | MR PRESSO | 10 | 2.894(0.819,10.22) | 0.133 |
| Hypertension | IVW | 11 | 0.726(0.140,3.760) | 0.703 |
|  | Weighted median | 11 | 0.939(0.205,4.299) | 0.935 |
|  | MR Egger | 11 | 0.867(0.001,924.901) | 0.969 |
|  | MR PRESSO | 9 | 0.717(0.191,2.696) | 0.636 |
| Stroke | IVW | 11 | 3.154(1.070,9.298) | 0.037 |
|  | Weighted median | 11 | 2.241(0.489,10.268) | 0.299 |
|  | MR Egger | 11 | 0.165(0.003,9.362) | 0.405 |
|  | MR PRESSO | 11 | 3.154(0.948,10.496) | 0.091 |
| Ischemic stroke | IVW | 11 | 3.736(1.185,11.782) | 0.024 |
|  | Weighted median | 11 | 2.891(0.543,15.383) | 0.213 |
|  | MR Egger | 11 | 0.465(0.004,49.848) | 0.756 |
|  | MR PRESSO | 11 | 3.736(1.021,13.670) | 0.074 |
| Large-artery stroke | IVW | 11 | 2.369(0.139,40.372) | 0.551 |
|  | Weighted median | 11 | 2.153(0.037,126.021) | 0.712 |
|  | MR Egger | 11 | 0.042(0.000,1142.76) | 0.557 |
|  | MR PRESSO | 11 | 2.369(0.141,39.735) | 0.562 |
| Small-vessel stroke | IVW | 11 | 5.756(0.411,80.667) | 0.194 |
|  | Weighted median | 11 | 8.173(0.241,277.47) | 0.243 |
|  | MR Egger | 11 | 63.499(0.005,7.61E5) | 0.409 |
|  | MR PRESSO | 11 | 5.756(0.718,46.146) | 0.130 |
| Cardioembolic stroke | IVW | 11 | 1.423(0.159,12.756) | 0.753 |
|  | Weighted median | 11 | 1.627(0.098,27.096) | 0.735 |
|  | MR Egger | 11 | 1.175(0,301,3.101) | 0.969 |
|  | MR PRESSO | 11 | 1.423(0.259,7.803) | 0.693 |

Supplementary Table 12 MR estimating the associations of biscuit cereal with mediator

| Outcome | Method | No. of SNPs | Beta (95% CI) | P value |
| --- | --- | --- | --- | --- |
| LDL-C | IVW | 5 | 0.332(-1.081,1.745) | 0.645 |
|  | Weighted median | 5 | 0.224(-1.503,1.952) | 0.799 |
|  | MR Egger | 5 | -0.536(-3.331,2.259) | 0.732 |
|  | MR PRESSO | 5 | 0.332(-0.473,1.137) | 0.464 |
| HDL-C | IVW | 5 | -0.832(-2.247,0.583) | 0.249 |
|  | Weighted median | 5 | -0.662(-2.436,1.113) | 0.465 |
|  | MR Egger | 5 | -0.175(-2.971,2.62) | 0.910 |
|  | MR PRESSO | 5 | -0.832(-2.058,0.394) | 0.254 |
| TC | IVW | 5 | 0.110(-1.307,1.527) | 0.879 |
|  | Weighted median | 5 | 0.330(-1.461,2.120) | 0.718 |
|  | MR Egger | 5 | -0.396(-3.197,2.405) | 0.800 |
|  | MR PRESSO | 5 | 0.110(-0.755,0.975) | 0.815 |
| apA-I | IVW | 5 | -0.495(-1.968,0.977) | 0.510 |
|  | Weighted median | 5 | -0.477(-2.341,1.388) | 0.616 |
|  | MR Egger | 5 | -0.408(-3.269,2.454) | 0.798 |
|  | MR PRESSO | 5 | -0.495(-0.809,-0.181) | 0.036 |
| apB | IVW | 5 | 1.107(-0.336,2.551) | 0.133 |
|  | Weighted median | 5 | 0.952(-1.007,2.910) | 0.341 |
|  | MR Egger | 5 | -0.200(-3.055,2.654) | 0.899 |
|  | MR PRESSO | 5 | 1.107(-0.125,2.339) | 0.153 |
| HbA1c | IVW | 5 | 0.126(-0.935,1.187) | 0.816 |
|  | Weighted median | 5 | -0.147(-1.448,1.154) | 0.825 |
|  | MR Egger | 5 | 1.111(-1.999,4.221) | 0.534 |
|  | MR PRESSO | 5 | 0.126(-0.430,0.682) | 0.680 |
| FG | IVW | 5 | 0.079(-0.234,0.392) | 0.622 |
|  | Weighted median | 5 | 0.164(-0.289,0.618) | 0.477 |
|  | MR Egger | 5 | -0.398(-1.553,0.757) | 0.548 |
|  | MR PRESSO | 5 | 0.079(-0.308,0.466) | 0.710 |
| FI | IVW | 5 | 0.346(0.008,0.684) | 0.045 |
|  | Weighted median | 5 | 0.388(-0.074,0.851) | 0.100 |
|  | MR Egger | 5 | 0.825(-0.086,1.735) | 0.174 |
|  | MR PRESSO | 5 | 0.346(-0.005,0.697) | 0.125 |
| BMI | IVW | 5 | 0.666(0.194,1.138) | 0.006 |
|  | Weighted median | 5 | 0.738(0.067,1.410) | 0.031 |
|  | MR Egger | 5 | -0.075(-1.547,1.396) | 0.926 |
|  | MR PRESSO | 5 | 0.666(0.159,1.173) | 0.062 |

Supplementary Table 13 MR estimating the associations of muesli with mediator

| Outcome | Method | No. of SNPs | Beta (95% CI) | P value |
| --- | --- | --- | --- | --- |
| LDL-C | IVW | 5 | -1.660(-3.152,-0.167) | 0.029 |
|  | Weighted median | 5 | -1.533(-3.422,0.357) | 0.112 |
|  | MR Egger | 5 | -3.101(-10.585,4.383) | 0.476 |
|  | MR PRESSO | 5 | -1.660(-2.677,-0.642) | 0.033 |
| HDL-C | IVW | 5 | -0.536(-2.032,0.959) | 0.482 |
|  | Weighted median | 5 | -0.428(-2.188,1.332) | 0.634 |
|  | MR Egger | 5 | -3.133(-10.627,4.362) | 0.473 |
|  | MR PRESSO | 5 | -0.536(-1.437,0.364) | 0.308 |
| TC | IVW | 5 | -1.127(-2.651,0.397) | 0.147 |
|  | Weighted median | 5 | -1.377(-3.302,0.548) | 0.161 |
|  | MR Egger | 5 | -2.503(-10.110,5.105) | 0.565 |
|  | MR PRESSO | 5 | -1.127(-2.158,-0.095) | 0.099 |
| apA-I | IVW | 5 | -0.440(-2.017,1.137) | 0.584 |
|  | Weighted median | 5 | -0.266(-2.189,1.656) | 0.786 |
|  | MR Egger | 5 | -2.565(-10.365,5.236) | 0.565 |
|  | MR PRESSO | 5 | -0.440(-1.550,0.670) | 0.480 |
| apB | IVW | 5 | -1.116(-2.642,0.410) | 0.152 |
|  | Weighted median | 5 | -0.778(-2.598,1.042) | 0.402 |
|  | MR Egger | 5 | -0.355(-8.007,7.298) | 0.933 |
|  | MR PRESSO | 5 | -1.116(-2.141,-0.091) | 0.100 |
| HbA1c | IVW | 4 | -1.229(-2.293,-0.165) | 0.024 |
|  | Weighted median | 4 | -0.909(-2.182,0.364) | 0.162 |
|  | MR Egger | 4 | -2.179(-12.442,8.084) | 0.718 |
|  | MR PRESSO | 4 | -1.229(-2.872,0.415) | 0.239 |
| FG | IVW | 5 | 0.139(-0.137,0.416) | 0.323 |
|  | Weighted median | 5 | 0.148(-0.211,0.507) | 0.419 |
|  | MR Egger | 5 | 1.467(0.035,2.899) | 0.138 |
|  | MR PRESSO | 5 | 0.139(-0.148,0.427) | 0.396 |
| FI | IVW | 5 | -0.0004(-0.308,0.307) | 0.998 |
|  | Weighted median | 5 | 0.028(-0.388,0.444) | 0.895 |
|  | MR Egger | 5 | 1.827(0.238,3.416) | 0.110 |
|  | MR PRESSO | 5 | -0.0004(-0.421,0.420) | 0.999 |
| BMI | IVW | 5 | -0.699(-1.117,-0.280) | 0.001 |
|  | Weighted median | 5 | -0.848(-1.434,-0.263) | 0.005 |
|  | MR Egger | 5 | -2.771(-5.034,-0.508) | 0.096 |
|  | MR PRESSO | 5 | -0.699(-1.264,-0.133) | 0.073 |

Supplementary Table 14 MR estimating the associations of other cereal with mediator

| Outcome | Method | No. of SNPs | Beta (95% CI) | P value |
| --- | --- | --- | --- | --- |
| LDL-C | IVW | 10 | -0.123(-1.260,1.015) | 0.833 |
|  | Weighted median | 10 | -0.113(-1.543,1.317) | 0.877 |
|  | MR Egger | 10 | -0.337(-4.102,3.428) | 0.865 |
|  | MR PRESSO | 10 | -0.123(-0.648,0.403) | 0.658 |
| HDL-C | IVW | 10 | -0.765(-1.905,0.374) | 0.188 |
|  | Weighted median | 10 | -0.425(-1.921,1.071) | 0.578 |
|  | MR Egger | 10 | 0.466(-3.306,4.237) | 0.815 |
|  | MR PRESSO | 10 | -0.765(-1.69,0.160) | 0.139 |
| TC | IVW | 10 | -0.071(-1.223,1.081) | 0.904 |
|  | Weighted median | 10 | -0.077(-1.567,1.414) | 0.919 |
|  | MR Egger | 10 | -0.756(-4.529,3.017) | 0.705 |
|  | MR PRESSO | 10 | -0.071(-0.934,0.792) | 0.876 |
| apA-I | IVW | 10 | -0.990(-2.188,0.208) | 0.105 |
|  | Weighted median | 10 | -0.866(-2.533,0.800) | 0.308 |
|  | MR Egger | 10 | 0.593(-4.101,5.287) | 0.811 |
|  | MR PRESSO | 10 | -0.990(-2.385,0.405) | 0.198 |
| apB | IVW | 10 | 0.285(-0.877,1.448) | 0.630 |
|  | Weighted median | 10 | 0.073(-1.372,1.519) | 0.921 |
|  | MR Egger | 10 | 0.344(-3.501,4.190) | 0.865 |
|  | MR PRESSO | 10 | 0.285(-0.311,0.882) | 0.373 |
| HbA1c | IVW | 13 | 1.013(0.389,1.636) | 0.001 |
|  | Weighted median | 13 | 1.208(0.249,2.166) | 0.014 |
|  | MR Egger | 13 | 3.004(0.516,5.493) | 0.037 |
|  | MR PRESSO | 13 | 1.013(0.200,1.826) | 0.031 |
| FG | IVW | 12 | 0.193(-0.004,0.39) | 0.055 |
|  | Weighted median | 12 | 0.207(-0.07,0.483) | 0.143 |
|  | MR Egger | 12 | 0.700(-0.092,1.493) | 0.114 |
|  | MR PRESSO | 12 | 0.193(0.021,0.365) | 0.050 |
| FI | IVW | 12 | 0.009(-0.323,0.340) | 0.959 |
|  | Weighted median | 12 | 0.004(-0.349,0.358) | 0.980 |
|  | MR Egger | 12 | -1.026(-2.256,0.204) | 0.133 |
|  | MR PRESSO | 12 | 0.009(-0.323,0.340) | 0.959 |
| BMI | IVW | 11 | 0.463(0.158,0.768) | 0.003 |
|  | Weighted median | 11 | 0.347(-0.088,0.781) | 0.118 |
|  | MR Egger | 11 | 0.589(-0.547,1.724) | 0.336 |
|  | MR PRESSO | 11 | 0.463(0.126,0.800) | 0.022 |

Supplementary Table 15 Heterogeneity and pleiotropy tests for the associations between Cereal intake and type and cardiovascular diseases

| exposure | Outcome | SNP  (number) | Heterogeneity test | | | Pleiotropy test | | | Global Test |
| --- | --- | --- | --- | --- | --- | --- | --- | --- | --- |
|  |  |  | Q | Q_df | Q -pval | Egger_  intercept | SE | p |  |
| Cereal intake | CVD | 32 | 38.451 | 31 | 0.168 | -0.012 | 0.012 | 0.306 | 0.163 |
| Cereal intake | MI | 32 | 34.763 | 31 | 0.293 | -0.010 | 0.012 | 0.402 | 0.299 |
| Cereal intake | HF | 32 | 51.615 | 31 | 0.011 | -0.013 | 0.011 | 0.251 | 0.015 |
| Cereal intake | HTN | 31 | 59.508 | 30 | 0.001 | -0.014 | 0.014 | 0.304 | 0.108 |
| Cereal intake | Stroke | 32 | 35.045 | 31 | 0.282 | -0.019 | 0.010 | 0.079 | 0.267 |
| Cereal intake | IS | 32 | 34.147 | 31 | 0.319 | -0.020 | 0.011 | 0.080 | 0.320 |
| Cereal intake | ISla | 32 | 41.021 | 31 | 0.108 | -0.054 | 0.030 | 0.083 | 0.108 |
| Cereal intake | ISsv | 32 | 22.463 | 31 | 0.868 | -0.021 | 0.025 | 0.400 | 0.871 |
| Cereal intake | ISce | 32 | 47.125 | 31 | 0.032 | -0.012 | 0.026 | 0.641 | 0.037 |
| Bran cereal | CVD | 12 | 9.473 | 11 | 0.578 | -0.002 | 0.010 | 0.812 | 0.603 |
| Bran cereal | MI | 13 | 10.146 | 12 | 0.603 | -0.0002 | 0.009 | 0.982 | 0.632 |
| Bran cereal | HF | 12 | 8.733 | 11 | 0.647 | -0.011 | 0.008 | 0.214 | 0.642 |
| Bran cereal | HTN | 13 | 10.234 | 12 | 0.595 | 0.014 | 0.009 | 0.134 | 0.590 |
| Bran cereal | Stroke | 12 | 18.251 | 11 | 0.076 | -0.002 | 0.013 | 0.898 | 0.077 |
| Bran cereal | IS | 12 | 23.768 | 11 | 0.014 | -0.006 | 0.016 | 0.706 | 0.019 |
| Bran cereal | ISla | 12 | 15.494 | 11 | 0.161 | 0.002 | 0.033 | 0.942 | 0.179 |
| Bran cereal | ISsv | 12 | 12.387 | 11 | 0.335 | -0.023 | 0.026 | 0.407 | 0.345 |
| Bran cereal | ISce | 12 | 23.333 | 11 | 0.016 | 0.014 | 0.032 | 0.667 | 0.019 |
| Biscuit cereal | CVD | 4 | 1.556 | 3 | 0.669 | 0.004 | 0.027 | 0.885 | 0.710 |
| Biscuit cereal | MI | 4 | 1.104 | 3 | 0.776 | 0.019 | 0.030 | 0.593 | 0.784 |
| Biscuit cereal | HF | 5 | 5.760 | 4 | 0.218 | 0.019 | 0.013 | 0.246 | 0.243 |
| Biscuit cereal | HTN | 5 | 4.812 | 4 | 0.307 | -0.006 | 0.012 | 0.676 | 0.452 |
| Biscuit cereal | Stroke | 5 | 5.769 | 4 | 0.217 | 0.027 | 0.017 | 0.215 | 0.296 |
| Biscuit cereal | IS | 5 | 8.272 | 4 | 0.082 | 0.047 | 0.018 | 0.082 | 0.135 |
| Biscuit cereal | ISla | 5 | 5.136 | 4 | 0.274 | 0.042 | 0.056 | 0.511 | 0.288 |
| Biscuit cereal | ISsv | 5 | 3.851 | 4 | 0.427 | 0.016 | 0.049 | 0.763 | 0.451 |
| Biscuit cereal | ISce | 5 | 13.601 | 4 | 0.009 | 0.084 | 0.048 | 0.181 | 0.021 |
| Oat cereal | CVD | 15 | 16.684 | 14 | 0.273 | -0.00005 | 0.010 | 0.996 | 0.284 |
| Oat cereal | MI | 16 | 13.640 | 15 | 0.553 | 0.001 | 0.009 | 0.931 | 0.580 |
| Oat cereal | HF | 15 | 26.103 | 14 | 0.025 | -0.005 | 0.011 | 0.628 | 0.035 |
| Oat cereal | HTN | 18 | 17.325 | 17 | 0.433 | 0.001 | 0.008 | 0.859 | 0.441 |
| Oat cereal | Stroke | 15 | 19.179 | 14 | 0.158 | -0.013 | 0.011 | 0.243 | 0.164 |
| Oat cereal | IS | 15 | 12.381 | 14 | 0.576 | -0.008 | 0.010 | 0.445 | 0.588 |
| Oat cereal | ISla | 15 | 15.723 | 14 | 0.331 | -0.021 | 0.027 | 0.441 | 0.323 |
| Oat cereal | ISsv | 15 | 15.515 | 14 | 0.344 | 0.023 | 0.024 | 0.362 | 0.327 |
| Oat cereal | ISce | 15 | 13.190 | 14 | 0.512 | -0.004 | 0.020 | 0.845 | 0.499 |
| Muesli | CVD | 5 | 6.617 | 4 | 0.158 | -0.069 | 0.027 | 0.085 | 0.238 |
| Muesli | MI | 5 | 4.247 | 4 | 0.374 | -0.027 | 0.032 | 0.452 | 0.462 |
| Muesli | HF | 5 | 3.496 | 4 | 0.478 | -0.028 | 0.022 | 0.294 | 0.528 |
| Muesli | HTN | 5 | 6.673 | 4 | 0.154 | -0.051 | 0.024 | 0.130 | 0.206 |
| Muesli | Stroke | 5 | 12.248 | 4 | 0.016 | 0.015 | 0.052 | 0.796 | 0.040 |
| Muesli | IS | 5 | 5.522 | 4 | 0.238 | 0.026 | 0.035 | 0.515 | 0.276 |
| Muesli | ISla | 5 | 4.245 | 4 | 0.374 | 0.062 | 0.077 | 0.478 | 0.427 |
| Muesli | ISsv | 5 | 0.712 | 4 | 0.950 | 0.041 | 0.066 | 0.578 | 0.950 |
| Muesli | ISce | 5 | 11.475 | 4 | 0.022 | 0.082 | 0.098 | 0.462 | 0.051 |
| Other cereal | CVD | 9 | 12.699 | 8 | 0.123 | 0.025 | 0.016 | 0.152 | 0.141 |
| Other cereal | MI | 9 | 8.338 | 8 | 0.401 | 0.025 | 0.015 | 0.131 | 0.418 |
| Other cereal | HF | 10 | 17.348 | 9 | 0.044 | -0.018 | 0.014 | 0.233 | 0.065 |
| Other cereal | HTN | 11 | 27.166 | 10 | 0.002 | -0.001 | 0.022 | 0.960 | 0.003 |
| Other cereal | Stroke | 11 | 12.367 | 10 | 0.261 | 0.019 | 0.013 | 0.170 | 0.286 |
| Other cereal | IS | 11 | 12.755 | 10 | 0.238 | 0.014 | 0.015 | 0.387 | 0.264 |
| Other cereal | ISla | 11 | 9.888 | 10 | 0.450 | 0.027 | 0.033 | 0.440 | 0.456 |
| Other cereal | ISsv | 11 | 6.217 | 10 | 0.797 | -0.016 | 0.030 | 0.614 | 0.814 |
| Other cereal | ISce | 11 | 6.021 | 10 | 0.814 | 0.001 | 0.025 | 0.961 | 0.831 |
| Muesli | LDL-C | 5 | 1.859 | 4 | 0.762 | 0.011 | 0.027 | 0.726 | 0.797 |
| Muesli | HDL-C | 5 | 1.450 | 4 | 0.835 | 0.019 | 0.027 | 0.538 | 0.851 |
| Muesli | TC | 5 | 1.832 | 4 | 0.767 | 0.010 | 0.028 | 0.742 | 0.785 |
| Muesli | apo-A-I | 5 | 1.981 | 4 | 0.739 | 0.016 | 0.029 | 0.624 | 0.751 |
| Muesli | apo-B | 5 | 1.804 | 4 | 0.772 | -0.006 | 0.028 | 0.855 | 0.780 |
| Muesli | HbA1c | 4 | 7.163 | 3 | 0.067 | 0.007 | 0.040 | 0.870 | 0.141 |
| Muesli | FG | 5 | 4.339 | 4 | 0.362 | -0.010 | 0.005 | 0.161 | 0.421 |
| Muesli | FI | 5 | 7.489 | 4 | 0.112 | -0.013 | 0.006 | 0.105 | 0.144 |
| Muesli | BMI | 5 | 7.300 | 4 | 0.121 | 0.015 | 0.008 | 0.164 | 0.207 |
| Other cereal | LDL-C | 10 | 1.919 | 9 | 0.993 | 0.001 | 0.012 | 0.910 | 0.993 |
| Other cereal | HDL-C | 10 | 5.928 | 9 | 0.747 | -0.008 | 0.012 | 0.521 | 0.777 |
| Other cereal | TC | 10 | 5.049 | 9 | 0.830 | 0.005 | 0.012 | 0.718 | 0.855 |
| Other cereal | apo-A-I | 10 | 12.209 | 9 | 0.202 | -0.011 | 0.016 | 0.507 | 0.246 |
| Other cereal | apo-B | 10 | 2.367 | 9 | 0.984 | 0.000 | 0.013 | 0.976 | 0.986 |
| Other cereal | HbA1c | 13 | 20.391 | 12 | 0.060 | -0.014 | 0.008 | 0.128 | 0.074 |
| Other cereal | FG | 12 | 8.395 | 11 | 0.678 | -0.003 | 0.003 | 0.224 | 0.685 |
| Other cereal | FI | 12 | 23.714 | 11 | 0.014 | 0.007 | 0.004 | 0.120 | 0.018 |
| Other cereal | BMI | 11 | 12.193 | 10 | 0.272 | -0.001 | 0.004 | 0.825 | 0.281 |
| Biscuit cereal | LDL-C | 5 | 1.298 | 4 | 0.862 | 0.008 | 0.012 | 0.531 | 0.845 |
| Biscuit cereal | HDL-C | 5 | 3.004 | 4 | 0.557 | -0.006 | 0.012 | 0.631 | 0.652 |
| Biscuit cereal | TC | 5 | 1.489 | 4 | 0.829 | 0.005 | 0.012 | 0.709 | 0.866 |
| Biscuit cereal | apo-A-I | 5 | 0.182 | 4 | 0.996 | -0.001 | 0.012 | 0.948 | 0.997 |
| Biscuit cereal | apo-B | 5 | 2.913 | 4 | 0.572 | 0.012 | 0.012 | 0.375 | 0.614 |
| Biscuit cereal | HbA1c | 5 | 1.099 | 4 | 0.894 | -0.007 | 0.011 | 0.556 | 0.899 |
| Biscuit cereal | FG | 5 | 6.115 | 4 | 0.191 | 0.003 | 0.004 | 0.452 | 0.223 |
| Biscuit cereal | FI | 5 | 4.310 | 4 | 0.366 | -0.004 | 0.003 | 0.348 | 0.411 |
| Biscuit cereal | BMI | 5 | 4.624 | 4 | 0.328 | 0.005 | 0.005 | 0.371 | 0.389 |


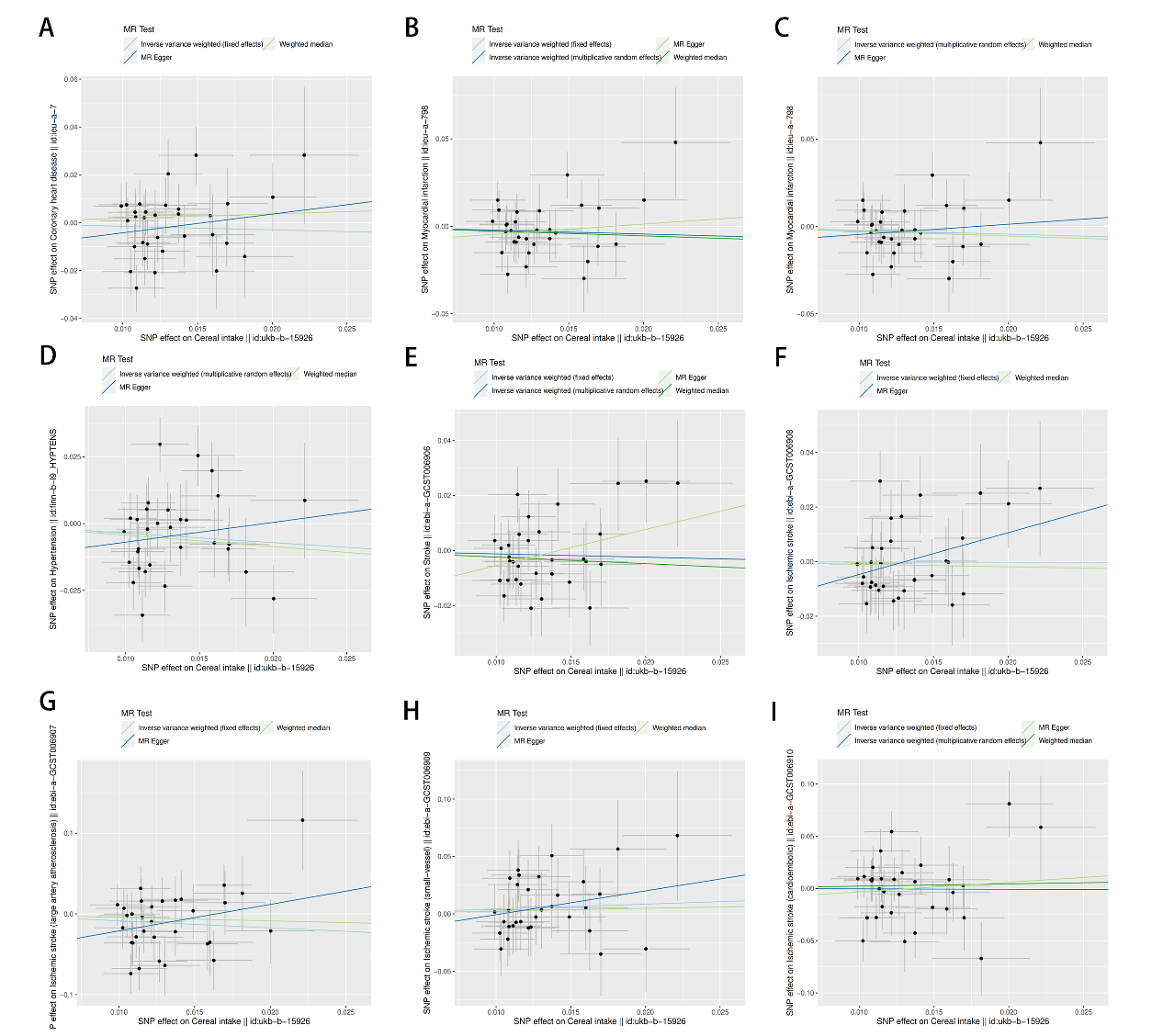


Supplementary Figure 1 Scatter plot using all IVs of cereal intake on CVDs


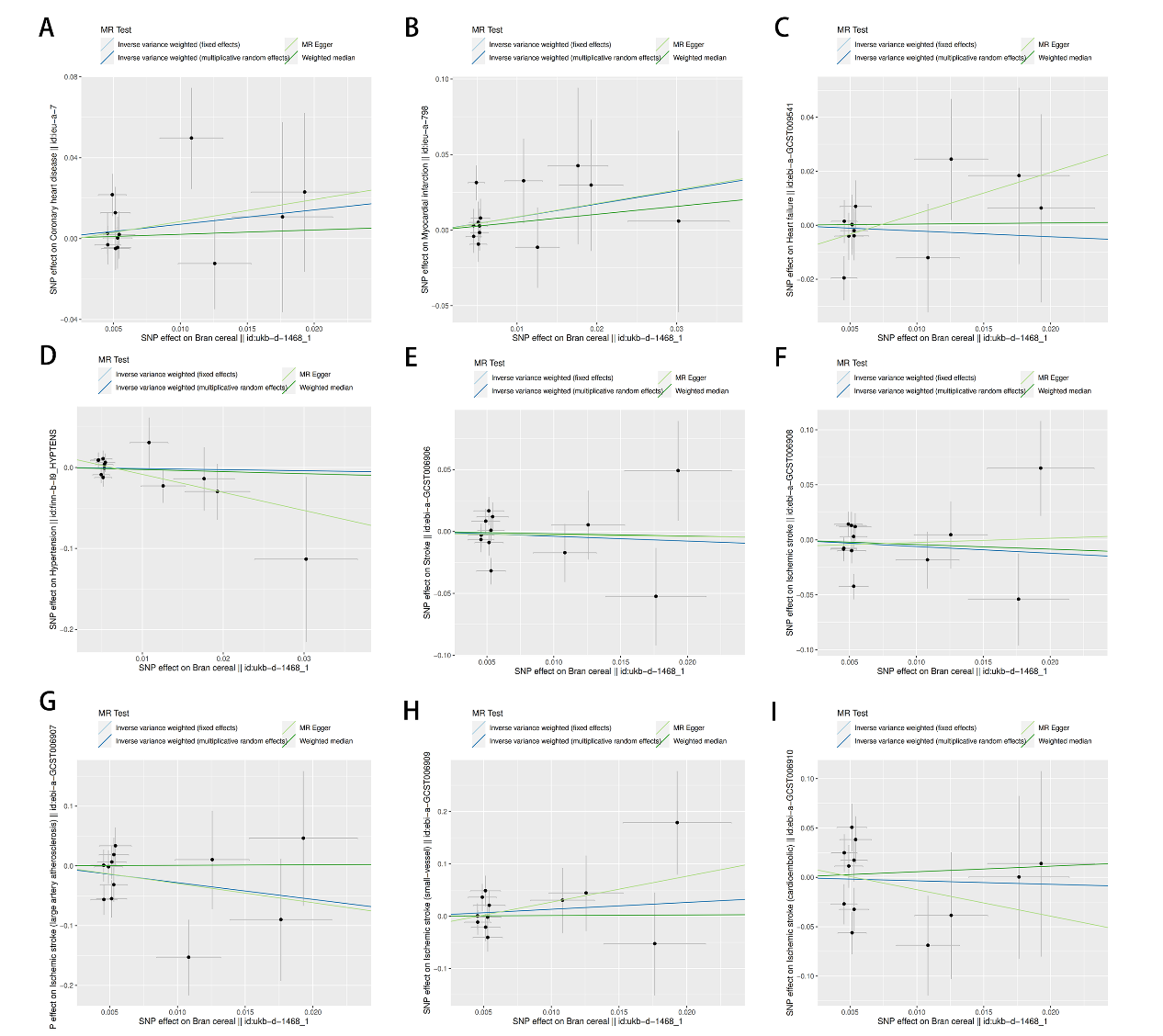


Supplementary Figure 2 Scatter plot using all IVs of bran cereal on CVDs


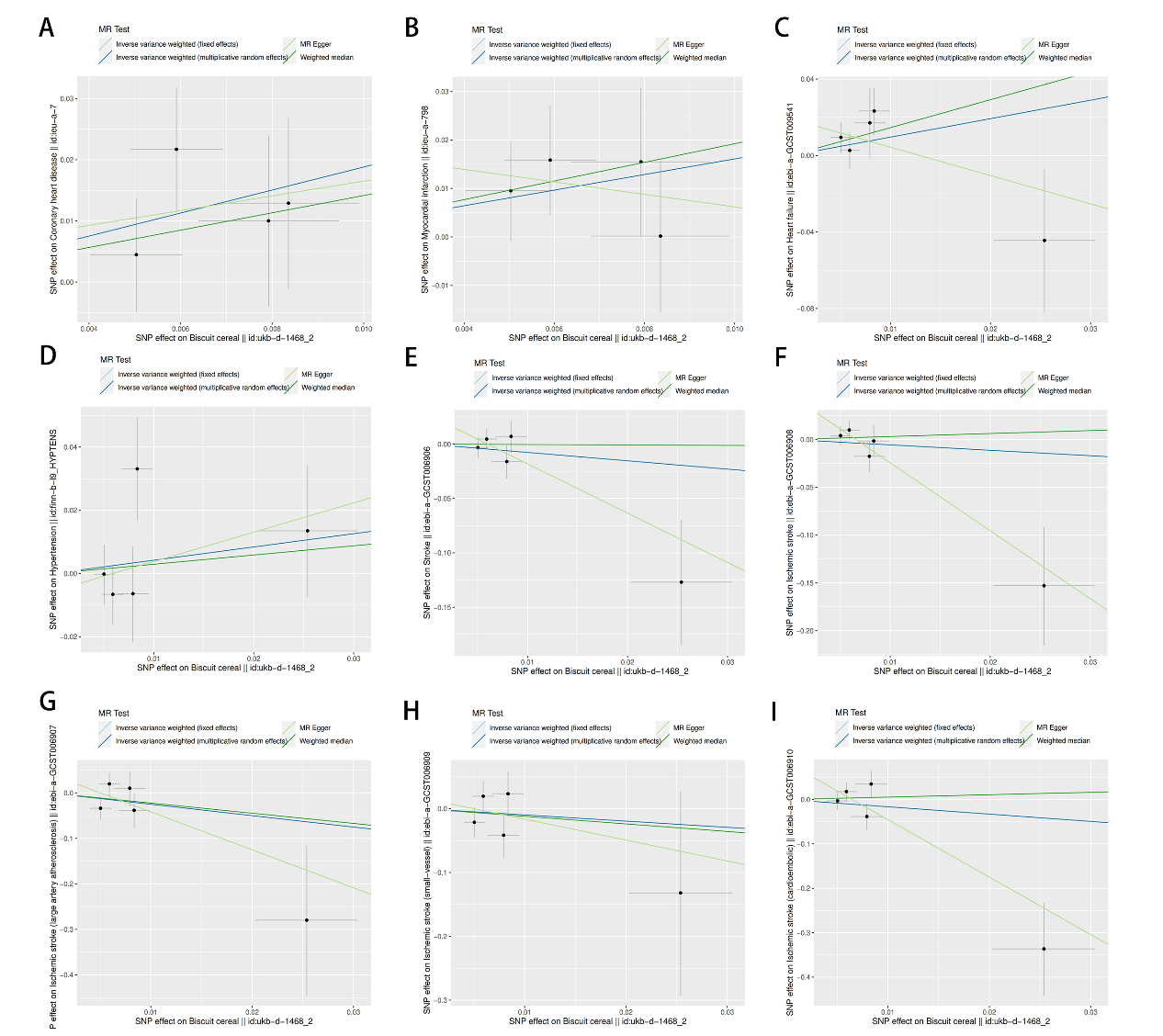


Supplementary Figure 3 Scatter plot using all IVs of biscuit cereal1 on CVDs


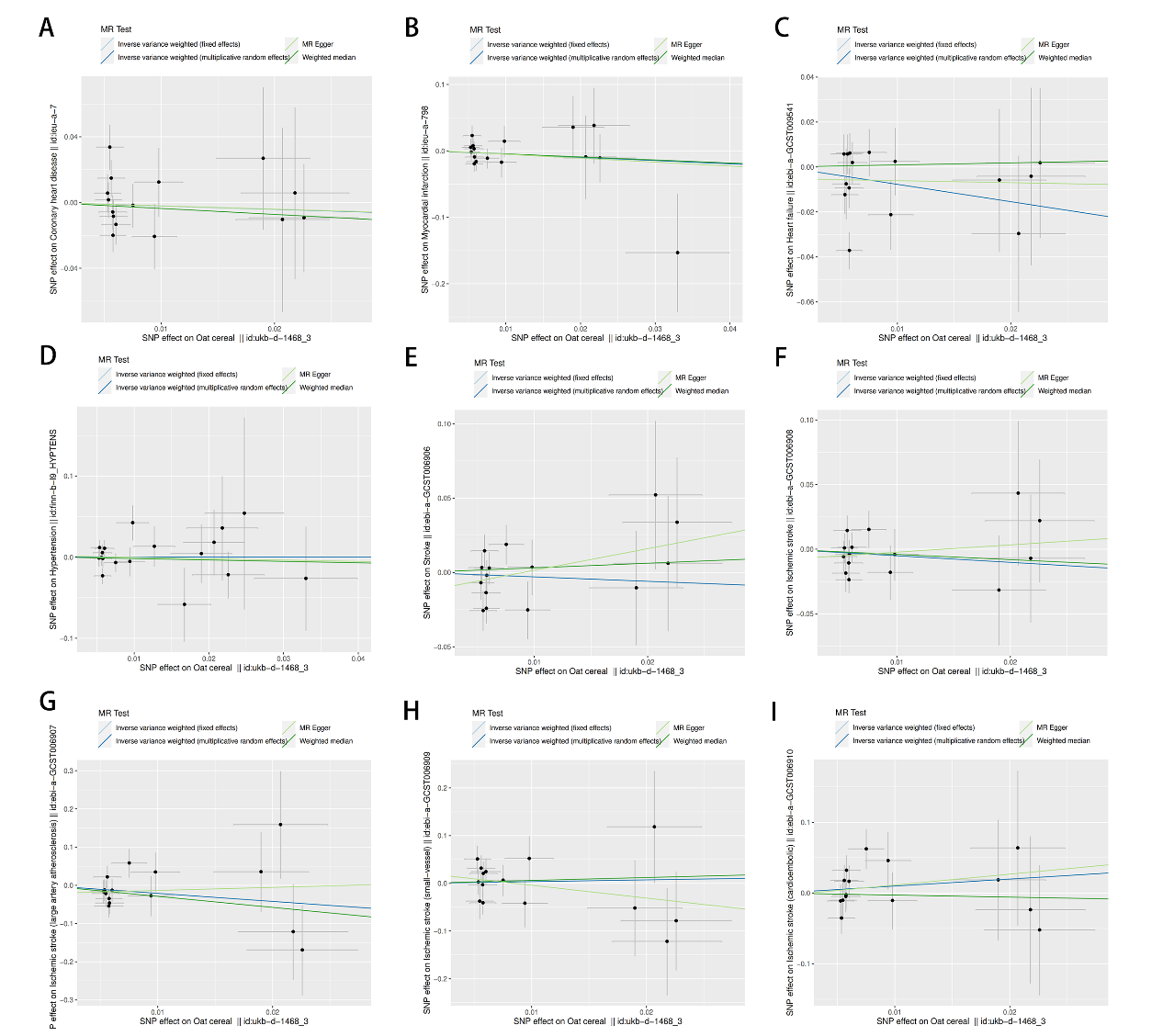


Supplementary Figure 4 Scatter plot using all IVs of oat cereal on CVDs.


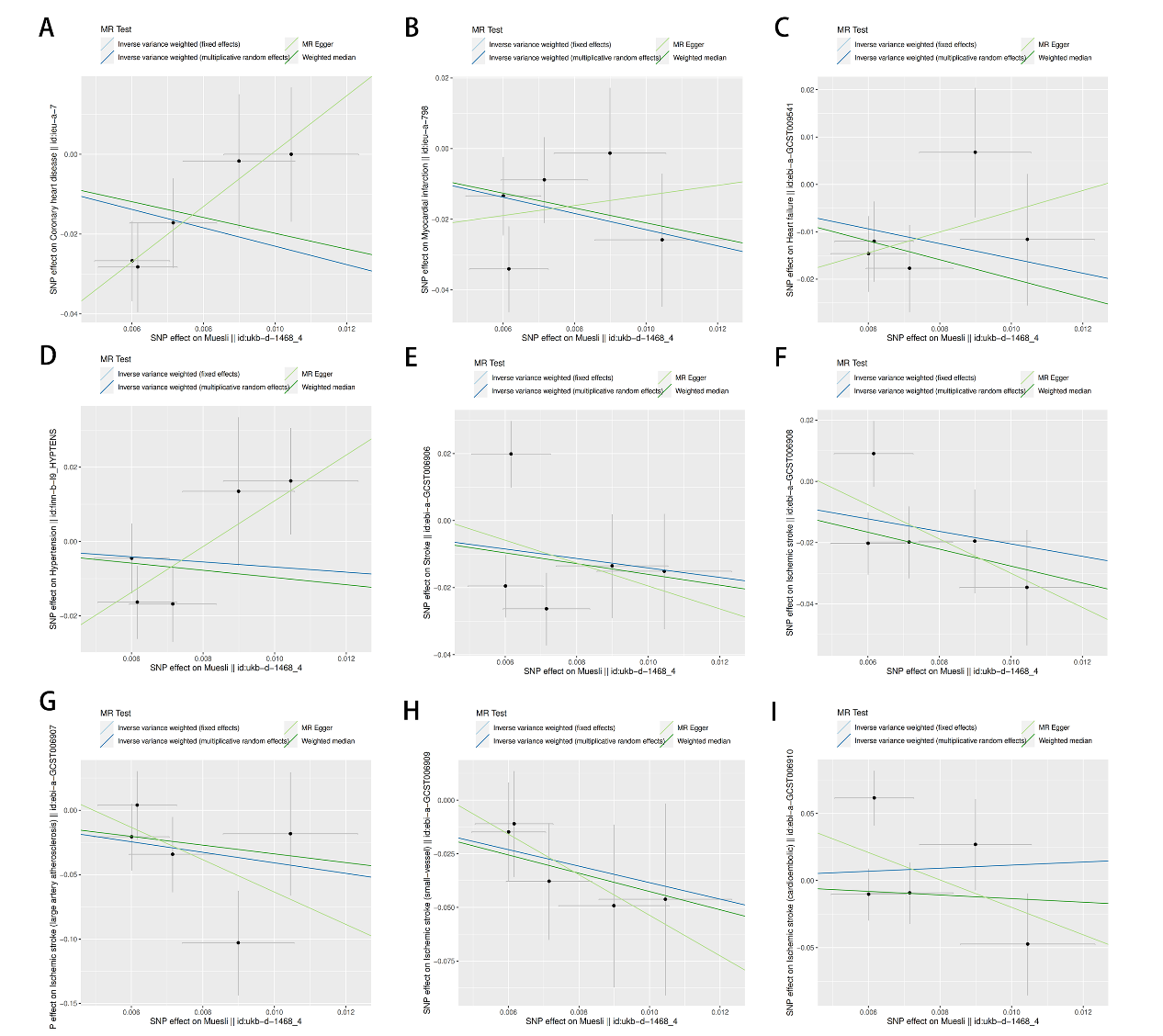


Supplementary Figure 5 Scatter plot using all IVs of muesli on CVDs.


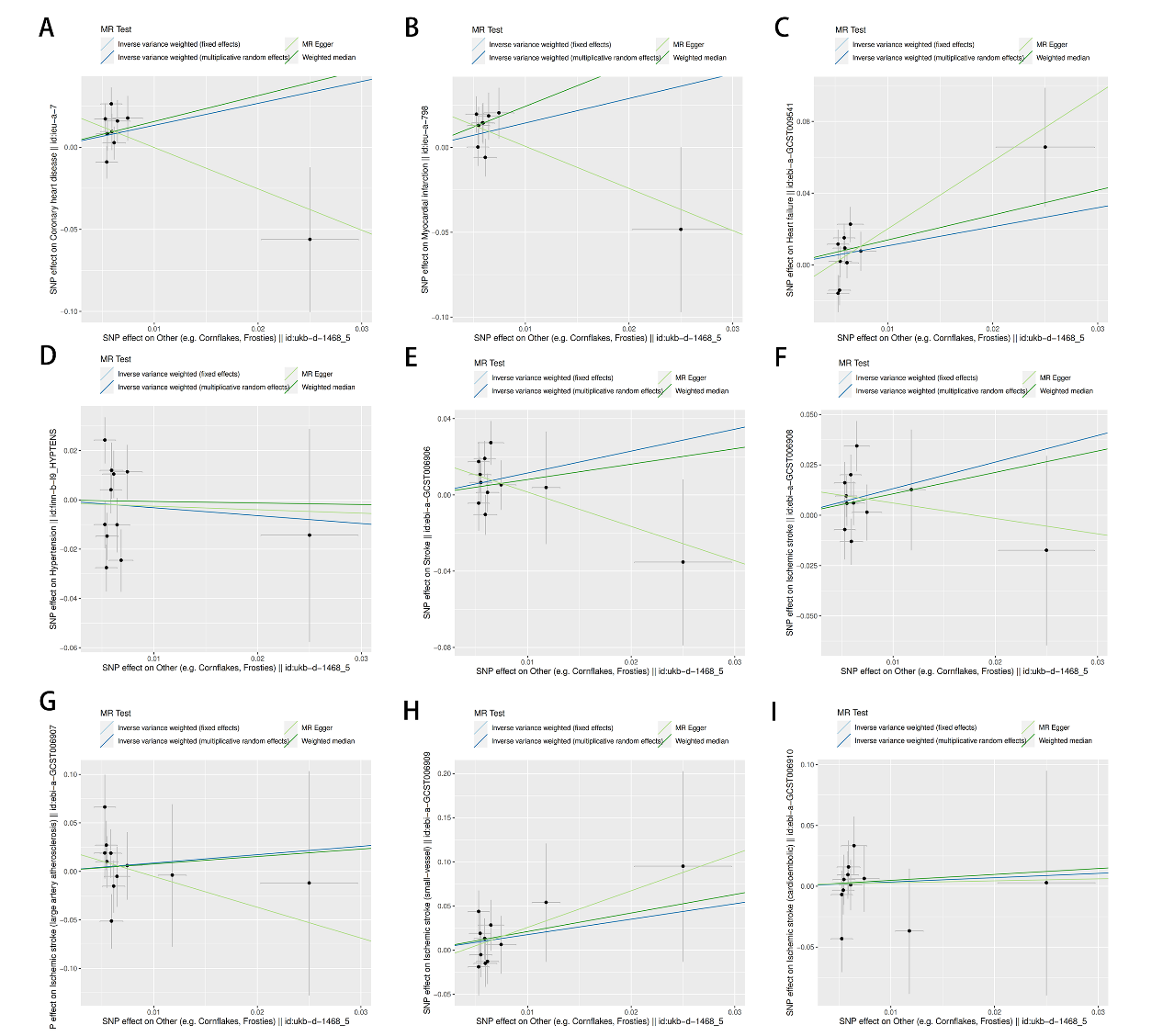


Supplementary Figure 6 Scatter plot using all IVs of other cereal on CVDs.


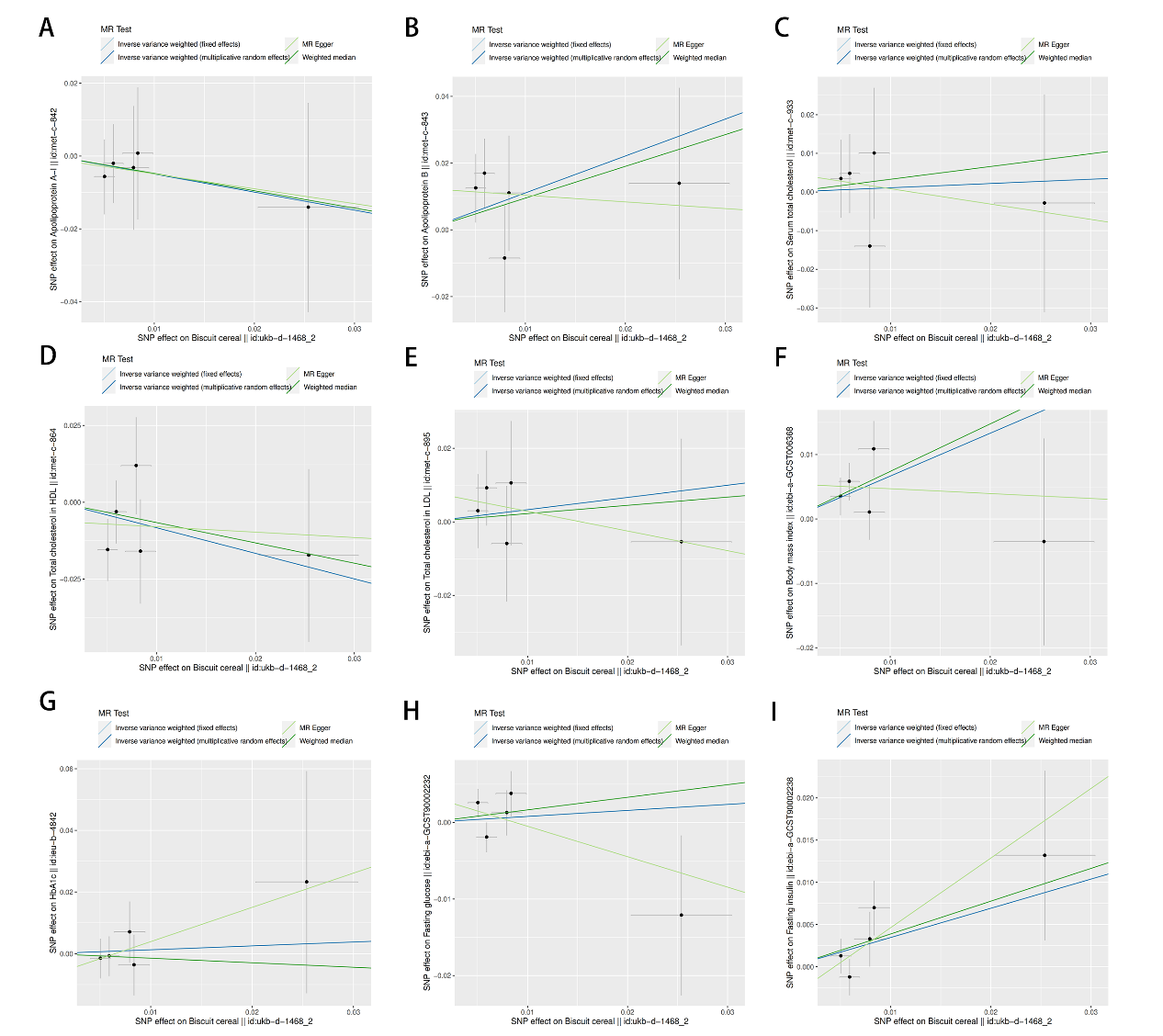


Supplementary Figure 7 Scatter plot using all IVs of biscuit cereal on CVD risk factors.


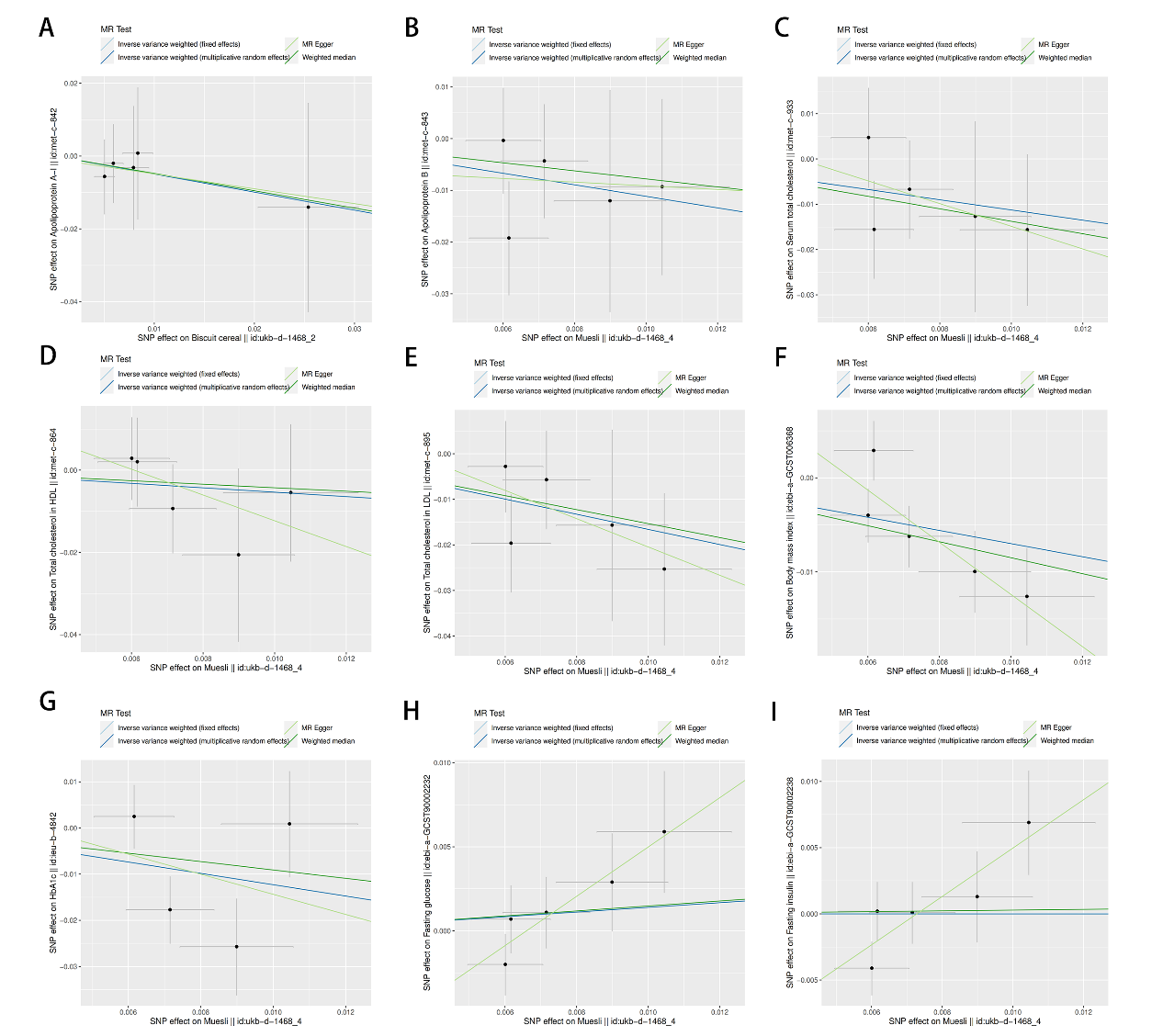


Supplementary Figure 8 Scatter plot using all IVs of muesli on CVD risk factors.


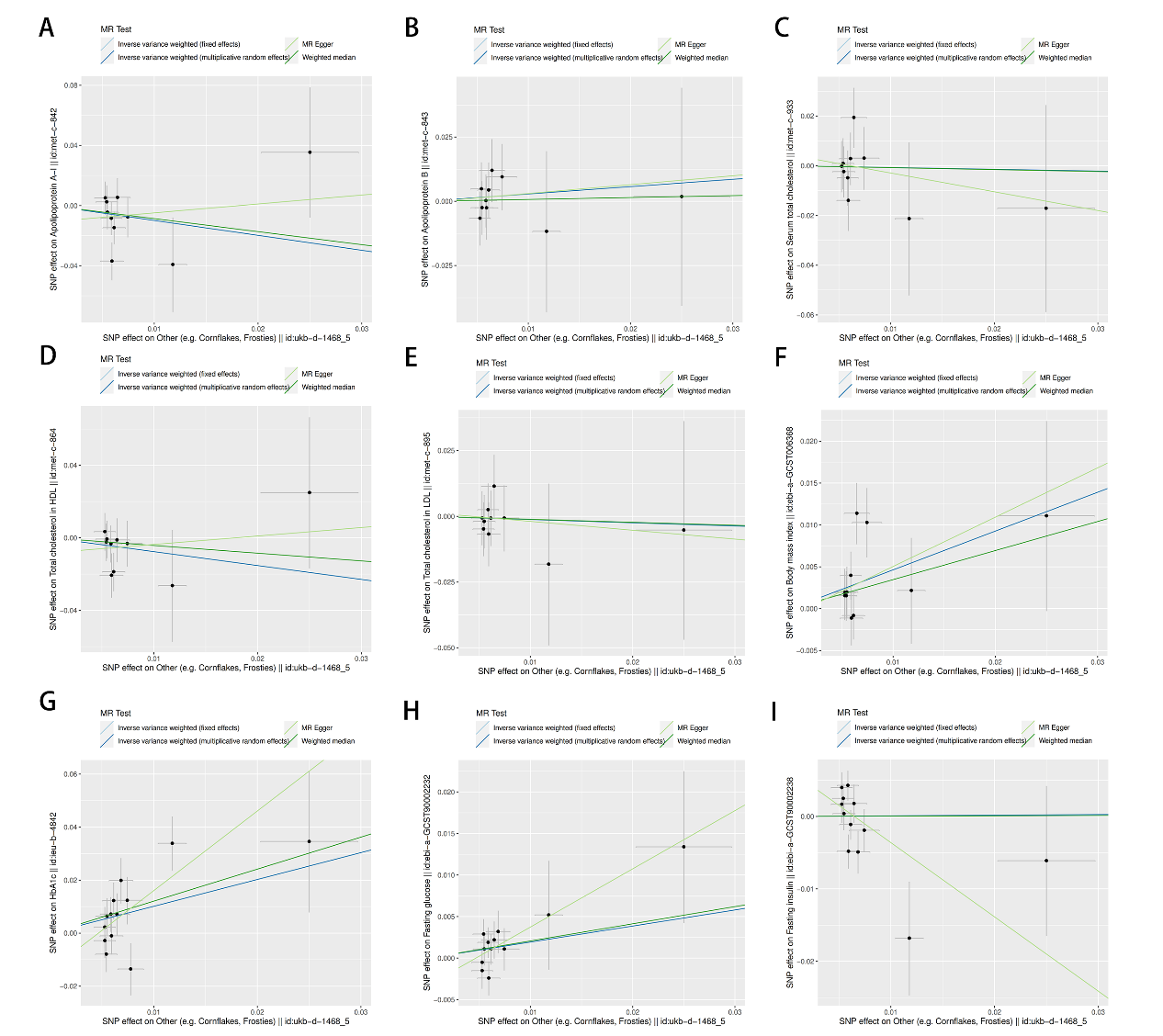


Supplementary Figure 9 Scatter plot using all IVs of other cereal on CVD risk factors.
